# West Nile virus infection and interferon alpha treatment alter the spectrum and the levels of coding and noncoding host RNAs secreted in extracellular vesicles

**DOI:** 10.1186/s12864-019-5835-6

**Published:** 2019-06-10

**Authors:** Andrii Slonchak, Brian Clarke, Jason Mackenzie, Alberto Anastacio Amarilla, Yin Xiang Setoh, Alexander A. Khromykh

**Affiliations:** 1 The Australian Infectious Diseases Centre, School of Chemistry and Molecular Biosciences, The University of Queensland, MBS building 76, Cooper Rd, St Lucia, QLD 4072 Australia; 2 The Pirbright Institute, Ash Rd, Pirbright, Surrey, GU24 GNF UK; 3 The Peter Doherty Institute for Infection and Immunity, Department of Microbiology and Immunology, The University of Melbourne, 792 Elizabeth Street, Melbourne, VIC 3000 Australia

**Keywords:** Extracellular vesicles, Exosomes, miRNA, West Nile virus, Flavivirus, snoRNA, Noncoding RNA

## Abstract

**Background:**

Extracellular vesicles (EVs) are small membrane vesicles secreted by the cells that mediate intercellular transfer of molecules and contribute to transduction of various signals. Viral infection and action of pro-inflammatory cytokines has been shown to alter molecular composition of EV content. Transfer of antiviral proteins by EVs is thought to contribute to the development of inflammation and antiviral state. Altered incorporation of selected host RNAs into EVs in response to infection has also been demonstrated for several viruses, but not for WNV. Considering the medical significance of flaviviruses and the importance of deeper knowledge about the mechanisms of flavivirus-host interactions we assessed the ability of West Nile virus (WNV) and type I interferon (IFN), the main cytokine regulating antiviral response to WNV, to alter the composition of EV RNA cargo.

**Results:**

We employed next generation sequencing to perform transcriptome-wide profiling of RNA cargo in EVs produced by cells infected with WNV or exposed to IFN-alpha. RNA profile of EVs secreted by uninfected cells was also determined and used as a reference. We found that WNV infection significantly changed the levels of certain host microRNAs (miRNAs), small noncoding RNAs (sncRNAs) and mRNAs incorporated into EVs. Treatment with IFN-alpha also altered miRNA and mRNA profiles in EV but had less profound effect on sncRNAs. Functional classification of RNAs differentially incorporated into EVs upon infection and in response to IFN-alpha treatment demonstrated association of enriched in EVs mRNAs and miRNAs with viral processes and pro-inflammatory pathways. Further analysis revealed that WNV infection and IFN-alpha treatment changed the levels of common and unique mRNAs and miRNAs in EVs and that IFN-dependent and IFN-independent processes are involved in regulation of RNA sorting into EVs during infection.

**Conclusions:**

WNV infection and IFN-alpha treatment alter the spectrum and the levels of mRNAs, miRNAs and sncRNAs in EVs. Differentially incorporated mRNAs and miRNAs in EVs produced in response to WNV infection and to IFN-alpha treatment are associated with viral processes and host response to infection. WNV infection affects composition of RNA cargo in EVs via IFN-dependent and IFN-independent mechanisms.

**Electronic supplementary material:**

The online version of this article (10.1186/s12864-019-5835-6) contains supplementary material, which is available to authorized users.

## Background

West Nile virus (WNV) is a member of Japanese encephalitis serogroup of *Flavivirus* genus, family *Flaviviridae,* which is distributed globally across temperate and tropical climates [1]. This is a small enveloped virus with ssRNA genome of positive polarity, which is capped, but not polyadenylated [2]. Viral genomic RNA encodes for 3 structural, 7 non-structural proteins and viral noncoding RNA (reviewed in [2–4]). WNV circulates between *Culex Sp.* mosquitos and birds but can also be transmitted to humans and horses that are the dead-end hosts [5]. WNV doesn’t have a specific tissue tropism in vertebrate host and can infect wide range of cells. After a mosquito bite virus first infects keratinocytes and Langerhans cells of skin. The later then migrate to lymph nodes, which are the sites of the initial virus replication. Infection then spreads systemically to visceral organs, where a second round of replication occurs primarily in epithelial cells. In some cases, WNV can cross the blood-brain barrier and infect neurons and glial cells [6]. WNV neuroinfection in humans results in encephalitis, meningitis and acute flaccid paralysis. In some cases, especially in aged and immunocompromised patients, WNV infection can be fatal or develop into serious long-term neurological abnormalities such as tandem gait, hearing loss, abnormal reflexes, and muscle weakness [7]. To date, no effective WNV therapies or vaccines are clinically available, and many facets of WNV molecular pathology remain uncharacterised [8].

Upon infection of the susceptible cells WNV activates a potent antiviral response that acts to prevent virus replication and spread to the neighbouring cells [9]. In vertebrate host it is triggered by pattern recognition receptors (PRRs), including retinoic-acid inducible gene-1 (RIG-I)-like receptors (RLR), Toll-like receptors (TLR), and the nucleotide oligomerization domain (Nod)-like receptors (NLR) [10]. They recognise molecular signatures of WNV infection such as dsRNA and activate transcription factors IRF3, IRF7, and NF-кB via the multicomponent signalling pathways. These factors activate expression of the genes encoding for pro-inflammatory cytokines and type I interferons (IFN-α/β) [11]. Once produced, IFN-α/β are secreted from the cells and can be transferred with blood flow. On the surface of acceptor cells IFN-α/β bind to the IFN-α/β receptor (IFNAR) and activate the JAK/STAT signalling cascade, which leads to transcriptional activation of hundreds of IFN-stimulated genes (ISGs) involved in establishing the innate antiviral immune response in infected and adjacent cells (reviewed in [12]).

For a long time, cell-to-cell signalling in response to WNV infection have been thought to be mediated by secreted type I IFN [13]. However, in the last decades, a novel mechanism of intercellular communication has been identified that involves intercellular signal transduction by extracellular vesicles (EVs) [14–17]. Extracellular vesicles are membrane-covered small vesicles secreted by the cells. They include apoptotic bodies produced by dying cells, microvesicles that result from shedding of plasma membrane and exosomes [18]. Exosomes are formed in the late endosomal compartment following inward budding of the endosomal membranes, which generates intracellular multivesicular bodies (MVB). MVBs contain large numbers of exosomes and release them following fusion of the MVB with the cellular plasma membrane [19]. Different types of extracellular vesicles can be distinguished by their size as exosomes have a diameter of 40-100 nm, microvesicles are 150-1000 nm in size and apoptotic bodies are 800-5000 nm being the largest extracellular vesicles [20, 21]. Exosomes can be also identified by specific biochemical markers associated with MVB such as CD9, CD63, CD81 and Hsp70, whereas microvesicles (MVs) can contain virtually any cellular protein [22]. Both exosomes [23] and MVs [24–28] can transfer their cargo to other cells and induce significant changes in their molecular processes. Molecular content of exosomes and MVs includes proteins [26, 29], mRNAs [24, 30] and miRNAs [23, 31–34]. Exosomes has been also reported to contain DNA [35, 36], circular RNA [37] and other types of noncoding RNAs [38]. It is believed that molecular cargo is sorted into exosomes in highly selective manner, whereas content of MVs represents a molecular “snapshot” of the cytoplasm [18].

Recent studies have highlighted the role of exosomes and MVs in the life cycle and pathogenesis of several RNA viruses (reviewed in [39, 40]), including transfer of the full-length genomic RNA of hepatitis C and hepatitis G viruses (Chivero et al. [41]; Dreux et al. [30]; Bukong et al. [42]; Ramakrishnaiah et al. [43]; Longatti et al. [44]), HIV-derived miRNAs [45], and antiviral protein APOBEC3G [46]. The role of EVs in infection with arboviruses, and particularly the members of flavivirus genus has not been investigated until recently, when exosomes produced by Dengue virus (DENV) infected cells were shown to transfer antiviral proteins IFITM3 and STING to promote antiviral response in neighbouring uninfected cells [17]. In addition, exosomes derived from Langat Virus (LGTV)-infected tick and human cells have been shown to transfer infectious viral RNA and non-structural proteins to uninfected cells [15]. Curiously, miRNAs and mRNAs related to antiviral response have been recently found in exosomes secreted by DENV-infected dendritic cells [47], however the effect of other flaviviruses on RNA profiles of EVs and the role of host-derived EV RNAs in flavivirus infection has not yet been investigated.

In order to fill this gap in knowledge we used next generation sequencing to characterize RNA content of EVs secreted by WNV-infected cells and found that infection significantly altered the composition of EV RNA cargo. Considering the role of IFN in regulation of antiviral signalling, we also assessed the ability of type I IFN to alter the levels of RNAs in EVs. We performed functional classification of mRNAs and miRNAs that exhibited the increased level in EVs and demonstrated their association with pro-inflammatory pathways and viral processes. In addition, we compared the effect of type I IFN treatment and of WNV infection on the RNA profiles of EVs and assessed the ability of WNV infection to alter incorporation of host RNAs into EVs in IFN-deficient cells. These analyses revealed that WNV-induced changes in profiles of RNAs in EVs are achieved via the combination of IFN-dependent and IFN-independent mechanisms.

## Results

### Elimination of WNV virions and RNA by heating and RNase treatment yields EV preparations free of infectious virus and viral RNA

Prior to RNA profiling, EVs free of cellular and viral material have to be isolated from culture fluid of infected cells. Isolation of EVs usually involves removal of cell debris and apoptotic bodies by centrifugation at 1000-4000 x g [48] followed by purification of EVs using ultracentrifugation, density gradient centrifugation, polyethylene glycol (PEG) precipitation, immunoaffinity binding, size exclusion chromatography or microfluidics sorting [49]. All these methods can more or less efficiently concentrate EVs and separate them from other components of the culture fluids such as serum and released cellular molecules [50]. However, due to the similar nature and biophysical properties of EVs and virions of enveloped viruses [51] removal of viral particles poses a significant challenge [52]. High-yield robust methods of EV isolation including ultracentrifugation and precipitation with PEG-based crowding agents have been reported to result in co-purification of viral particles. Gradient centrifugation and affinity purification of exosomes on antibody-coupled beads was shown to result in efficient virus-EVs separation, but application of these methods are limited due to a low yield of purified EV and lack of universal EV marker [20, 52].

Taking into consideration that virions of larger viruses e.g. retroviruses [53] and poxviruses [54] have been reported to incorporate host miRNAs, presence of viral particles in EV preparations can potentially affect the results of RNA profiling. Although flaviviruses have very densely packed virions [55, 56] of significantly smaller size (50nm WNV vs 100nm of HIV or 200x300nm of poxvirus) and therefore unlikely to have a capacity for carrying host RNAs, we attempted to obtain preparation of EVs that are free of viral particles. To overcome the issues and limitations associated with gradient centrifugation and affinity purification, we devised the approach that employs different thermal stability of viral particles and EVs to eliminate virus and viral RNAs from the culture fluids prior to isolation of EVs by PEG precipitation.

Heating to 65^o^C has been previously reported to completely inactivate hepatitis C virus (HCV) virions within 8 min [57] and heating for 30 min at 40^o^C has been shown to disintegrate dengue virus (DEN) virions causing them to physically burst and release their content [58]. EVs, however, are remarkably stable and physical challenges including heating, treatment with moderate concentrations of ethanol or pH changes has no effect on their integrity and content [59]. Therefore, heating of culture fluids should eliminate viral particles, while preserving EVs, which can then be purified by precipitation with PEG-based reagent.

**Fig. 1:**
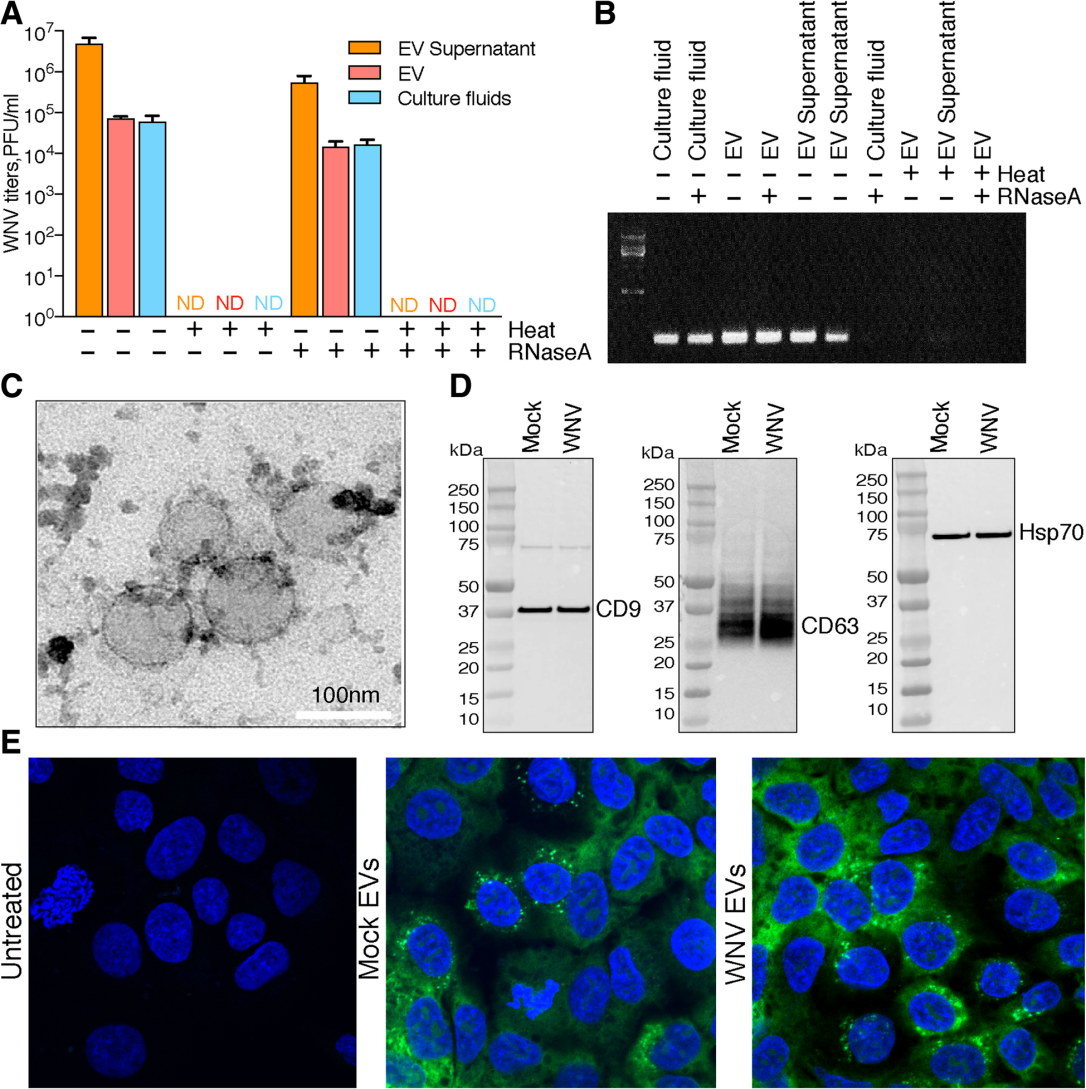
Isolation and characterization of extracellular vesicles. WNV titres (**a**) and detection of viral RNA by RT-PCR (**b**) in EV preparations before and after heat-inactivation and RNase A treatment. Culture fluids from WNV-infected were centrifuged to remove cell debris and incubated for 30min at 56^o^C with or without 1U/ml of RNaseA. EVs were then isolated from treated and untreated culture fluids using and analysed for the presence of infectious viral particles by plaque-assay on BHK-21 cells. **b** RT-PCR for viral RNA on sample of culture fluids and EV preparations **c** Transmission electron microscopy of EV preparations. **d** Western-blot analysis of EV lysates for the presence of exosomal markers. **e** Confocal microscopy analysis of A549 cells treated with Mock or WNV EVs containing fluorescently stained RNA cargo. Bright green fluorescence corresponds to SYTO RNASelect-stained EV RNA, blue fluorescence corresponds to DAPI-stained DNA. **e** Values in **a** represent the means of 4 biological replicates ±SD

To test if the exposure to the high temperature can selectively eliminate the virus, culture fluids from A459 cells infected with WNV at multiplicity of infection (MOI) = 1 were harvested at 24h post infection. Samples were centrifuged to remove cell debris and apoptotic bodies and cleared supernatants were incubated at 56^o^C for 30 min or at 4^o^C. The temperature was selected to be between the values used to inactivate DENV and HCV [57]. After the heat treatment samples were subjected to the additional round of centrifugation to remove potentially precipitated denatured serum proteins, and extracellular vesicles were then isolated from supernatants using ExoQuick TC reagent (PEG-based). PEG-based precipitation method was chosen for isolation of EVs because it is more reproducible than ultracentrifugation and gives higher yields [49, 50, 60]. EV-pellets and culture fluids before and after (supernatants in Fig. 1a, b) EV precipitation were tested for infectious virus by plaque-assay, which demonstrated that heating effectively removed infectious virus from the culture fluid and from the EV preparation (Fig. 1a). In addition, RNA was isolated from the collected material and analysed for the presence of viral genomic RNA by RT-PCR. The results showed presence of trace amounts of viral RNA in EV preparations after heating (Fig. 1b). To determine if this residual RNA represented RNA leaked from disintegrated virions or RNA contained within the viral particles that resisted incubation at high temperature, the ability of membrane vesicles to protect their RNA content from degradation by RNases [61, 62] was employed. Incubation of heat-treated culture fluids with RNase should eliminate extravesicular RNA, while having no effect of RNA contained within EVs and/or viral particles. Therefore, we performed heat treatment of EVs in the presence of RNaseA followed by RNA isolation and RT-PCR detection of viral RNA. RT-PCR analysis did not detect any viral RNA in EV pellets isolated from culture fluid following heat and RNase treatments (Fig. 1b), demonstrating that the residual RNA detected after heat treatment was indeed extravesicular and was completely removed by RNase digestion. Therefore, combination of heat and RNase treatment followed by PEG-precipitation yielded EVs that did not contain detectable amounts of WNV infectious particles and viral RNA.

To determine if EVs remained intact after heat and RNaseA treatment, the EV preparations were first analysed by transmission electron microscopy (TEM) and were found to contain vesicles with visually undamaged membranes (Fig. 1c). They were ~100 nm in diameter, which is the characteristic size of exosomes [20]. Vesicles of bigger size could not be detected by TEM, which suggest that EV preparation do not contain apoptotic bodies.

To further analyse the composition of EV preparations, lysates of EVs were subjected to Western blotting with antibodies specific to the exosomal markers. Western blot analysis demonstrated that EV preparations were positive for CD69, CD9 and Hsp70 and thus contained exosomes (Fig. 1d). However, we could not assess whether or not EV preparation contain MVs as they vary significantly in size and protein content and no specific biochemical markers have been so far assigned to MVs [20, 21]. Taking into consideration that all cell types tested to date were shown to secrete MVs and that they can co-purify with exosomes in PEG-precipitation [63], it is possible that our EV preparation could contain MVs in addition to exosomes and therefore we refer to the isolated particles using more general term “extacellular vesicles” or EVs to avoid confusion.

To assess if isolated EVs retained their RNA cargo, RNA content of extracellular vesicles isolated from culture fluids of WNV-infected and mock-infected cells was fluorescently labelled by RNA-specific dye SYTO RNASelect. Stained EVs were then used to treat uninfected A549 cells. At 24h after treatment cells were fixed and analysed by confocal microscopy. Bright green fluorescence of SYTO RNASelect-stained EV RNA was observed in cells treated with both, mock and WNV EVs (Fig. 1e), indicating that EV retained their RNA cargo after isolation procedure and can deliver it into acceptor cells.

Therefore, we established the procedure for isolation of the EVs from culture fluid of WNV-infected cells, which yielded intact EVs and preserved their RNA cargo, but eliminated infectious virions and viral RNA. This procedure was further used in the study to obtain EVs for the next generation sequencing analysis.

### WNV infection and IFN-alpha treatment alter levels of coding and noncoding host RNAs in extracellular vesicles

To investigate the effect of WNV infection on RNA profiles of EVs, A549 human alveolar carcinoma cells were infected with Kunjin strain of WNV at multiplicity of infection (MOI)=1. A549 cells were selected for the study because they are (i) of human origin, (ii) susceptible to infection with WNV and (iii) have been reported to develop a competent antiviral response [64]. After incubation with virus inoculum cells were washed and then maintained in exosomes-free culture media. Uninfected cells kept under the same conditions were used as a control. Taking into account that host response to WNV is most active at the early time point with the IFN secretion peaking around 24h post infection [65], culture fluids for isolation of EVs were harvested at 24h after infection to ensure that they contain the vesicles released during active phase of antiviral activity. In parallel we also sequenced and analysed RNA content of EVs isolated from culture fluids of A549 cells treated with IFN-alpha. Type I IFN has been shown to trigger incorporation of antiviral proteins into exosomes [46, 66] and we previously showed secretion of IFNa/b by A549 cells infected with WNV [67]. To ensure the consistency between studied conditions, Mock and IFN-treated cells were also allowed to release exosomes into culture media for 24h prior to collection of culture fluid. EVs were then purified from harvested culture fluids following the developed isolation protocol and used for RNA extraction. Although culture fluids from Mock-infected and IFN-treated cells do not contain viral particles, the same procedure with heating and RNase treatment was also used to isolate EVs from these samples to avoid the potential bias related to different sample processing.

**Fig. 2:**
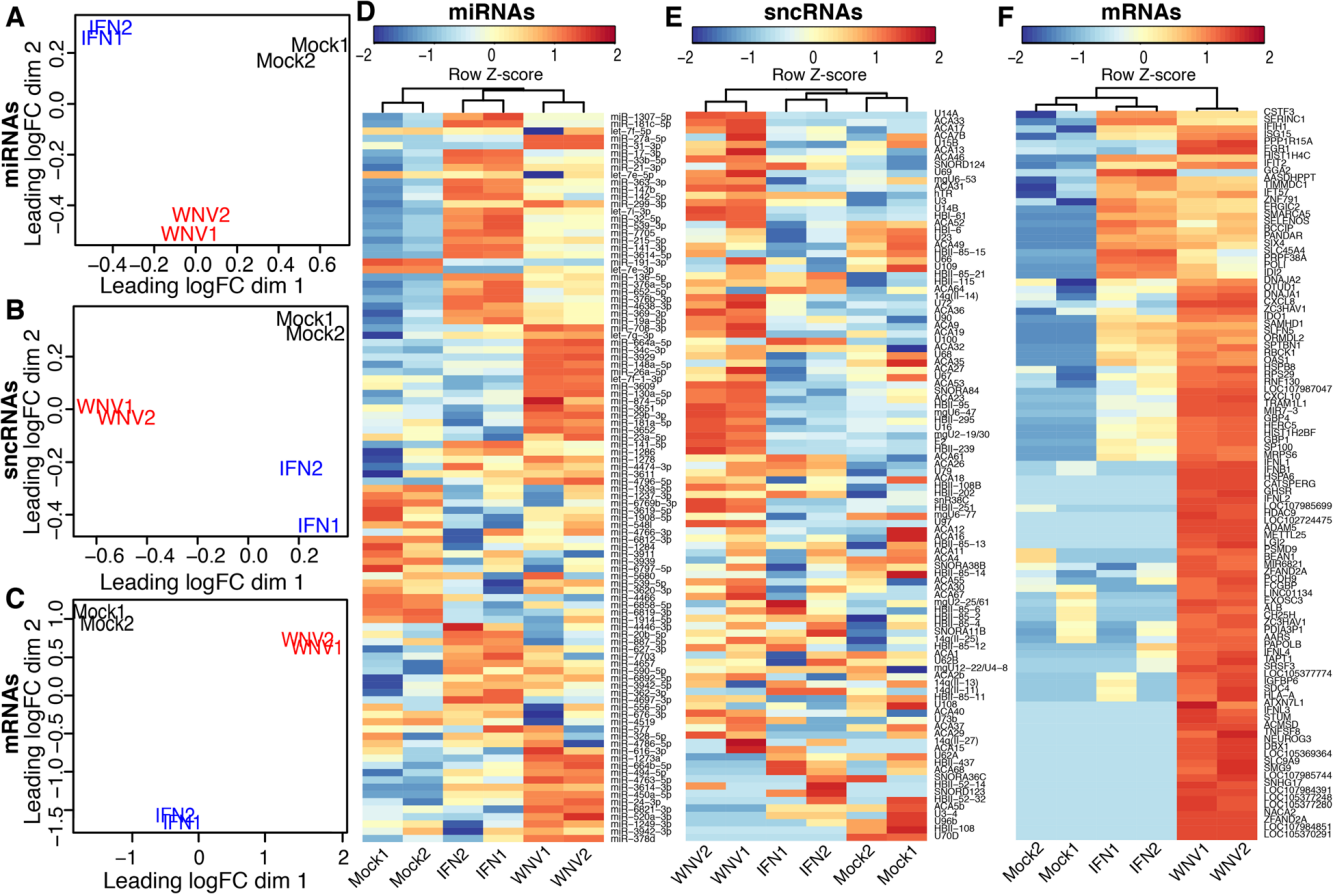
WNV infection and IFN-alpha treatment alter the profiles of coding and noncoding RNAs in EVs. Multi-dimensional scaling analysis (MDS) plots with two principal components based on the profiles of miRNAs (**a**), sncRNAs (**b**) and mRNAs (**c**) from EVs show distance between the samples. Hierarchical clustering and heat maps on RNA-Seq data reads mapped to human genome showing variations in the content of the top 100 most variable miRNAs (**d**), sncRNAs (**e**) and mRNAs (**f**) between the groups and biological replicates

Enriched small and long RNA fractions were then isolated from EVs and used to prepare cDNA libraries that were subsequently sequenced using Illumina NGS platform. High quality RNA-Seq reads were then mapped to human genome and levels of corresponding miRNAs, sncRNAs and mRNAs were quantified. The data quality control test showed high consistency between biological replicates in library compositions and abundance of each individual RNA for all samples except sncRNAs, that were somewhat more variable, but still within the confidence range for subsequent analysis (Additional file 1: Figure S1). After the quality test data exploration was performed on all datasets by multidimensional scaling (MDS) analysis and hierarchical clustering (Fig. 2). MDS analysis demonstrated that Mock, WNV and IFN EVs have different miRNA, sncRNAs and mRNAs content. Notably, a higher distance in dimension 1 was estimated for EV miRNAs between IFN-treated and Mock samples than between WNV-infected and Mock samples (Fig. 2a), suggesting that IFN treatment induces more profound changes in levels of miRNAs in EVs than WNV infection. However, for sncRNAs and mRNAs, larger distance was computed between WNV and Mock EV samples compared to that between IFN-treated and Mock EV samples, suggesting that WNV infection induces more profound changes in abundance of these RNAs in EVs than treatment with IFN (Fig. 2b, c). In addition, we noticed some separation between the biological replicates of sncRNA in IFN EVs, which explains variations previously observed in data quality analysis. All other biological replicates were positioned very close to each other on MDS plots indicating high consistency between replicates. In hierarchical clustering analysis, IFN-treated and WNV-infected samples of EV miRNAs were clustered together in a separate cluster from mock (Fig. 2d). This indicates that both treatments altered the profiles of miRNAs in EVs although IFN treatment altered miRNA levels to higher extend according to the MDS analysis. In line with the MDS results, hierarchical clustering revealed that WNV infection induced more pronounced changes in sncRNAs and mRNAs profiles compared to IFN treatment as IFN and Mock samples were clustered separately from WNV samples for these RNAs (Fig. 2e, f). Therefore, the results of data exploration suggest that WNV infection and IFN-alpha treatment induce distinctive changes in the spectrum and the levels of coding and noncoding RNAs in EVs.

### WNV infection and IFN-alpha treatment alter secretion in EVs of miRNAs that regulate host genes associated with viral processes and pro-inflammatory pathways

Regulatory small RNAs and particularly miRNAs have been previously shown to play a major role in cell-to-cell signal transduction by EVs in processes such as cancer progression and cells differentiation [68]. Recent studies also highlighted the role of EV-incorporated miRNAs in virus-host interactions and modulation of antiviral response (reviewed in [52, 69]).

**Fig. 3:**
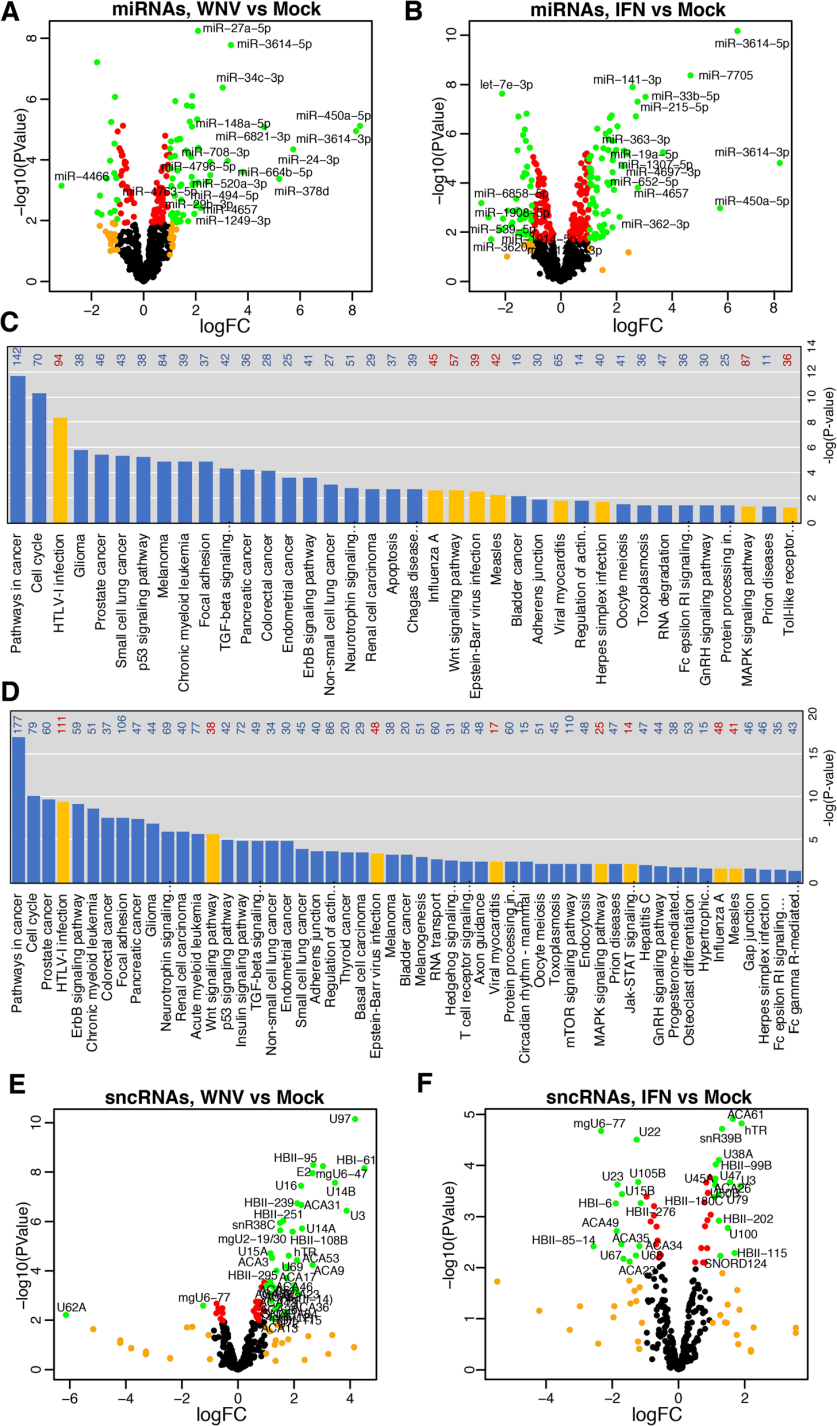
Analysis of small RNAs incorporated into EVs secreted by WNV-infected and IFN-treated cells. **a** and **b** Volcano plots demonstrating significance and fold change in abundance of individual miRNAs in EVs secreted from WNV-infected (**a**) and IFN-treated (**b**) A549 cells compared to mock. **c** and **d** Bar plots showing KEGG-pathways significantly enriched in genes targeted by miRNAs with increased abundance in EVs secreted from WNV-infected (**c**) or IFN-alpha-treated (**d**) cells. Pathways related to virus infection are shown in yellow; numbers at the right indicate the number of pathway-associated genes annotated as targets of EV miRNAs. **e** and **f**. Significance and logFC of sncRNAs in EVs from WNV-infected (**e**) and IFN-treated (**f**) A549 cells compared to mock is represented by the volcano plots. In (**a**, **b**, **e** and **f**) red colour indicates for RNAs exhibiting logFC>1, orange shows RNAs with FDR-adjusted *P*-values <0.05 and green indicates for RNAs with logFC>1 and *P*-Value<0.05 (selected for further analyses and functional annotation). RNAs with increased abundance are located on the right side ((+) LogFC values) and RNAs with decreased abundance are located on the left side ((-) LogFC values). Gene names are indicated for RNAs that exhibited the highest change in response to treatment/infection (*P*<0.001, logFC>2 for miRNAs and *P*<0.01, logFC>2 for sncRNAs)

To determine which miRNAs have different abundance in EVs secreted by WNV-infected and IFN-alpha treated cells, RNA-Seq data were subjected to the statistical analysis for differential miRNA expression. The analysis demonstrated that WNV infection significantly (FDR<0.05, Log2 fold change (LogFC) > 1) altered levels of 73 miRNAs (Fig. 3a, Additional file 4: Table S1). Amongst miRNAs with altered levels in WNV-infected EV samples 56 miRNAs had increased abundance and 17 had diminished levels compared to mock (Fig. 3a, Additional file 4: Table S1). All but one of the decreased miRNAs exhibited moderate changes in abundance (1-2 logFC) and were at the borderline of statistical significance, whereas increased miRNAs demonstrated up to 8 logFC increment (Fig. 3a) with high significance (FDR<0.001). IFN-alpha treatment changed the abundance of 115 miRNA (Fig. 3b, Additional file 4: Table S2). Levels of 72 miRNAs were increased in EVs secreted by IFN-treated cells, while levels of 53 miRNAs were decreased (Fig. 3b, Additional file 4: Table S2). The trend in direction of changes in EVs miRNA abundance after IFN treatment was similar to that observed after WNV infection – increased miRNAs had notably higher logFC (up to 8 log) than those that were decreased, which mostly showed less than 2 logFC (Fig. 3a, b). These results show that WNV infection and IFN treatment predominantly stimulate, rather than inhibit incorporation of specific host miRNAs into EVs.

MicroRNAs regulate the expression of target genes predominantly by binding to the complementary sites in 3’UTRs of mRNAs [70]. This usually results in translational repression [71], but can also lead to degradation of target mRNAs [72] or even stabilization of mRNAs [73] or up-regulation of their translation [74]. Therefore, identification of mRNAs that are targeted by miRNAs is required for prediction of miRNA functions. Thus, to determine the biological implication of miRNAs that showed increased abundance in EVs secreted by WNV-infected and IFN-treated cells, identification and functional classification of their mRNA targets was performed. For this analysis the lists of targets for all miRNAs with significant >1 log2 increase in WNV and IFN EVs were extracted from the curated database of experimentally validated miRNA targets. These lists were filtered for high-confidence interactions and used to reconstruct miRNA-mRNA networks. The association between the targets of miRNAs and KEGG pathways and relative weight of the targets (depends on number of interacting miRNAs) were then used to calculate the enrichment of miRNA targets in the biological pathways. The network analysis revealed that miRNAs with increased abundance in EVs secreted from WNV-infected and IFN-alpha-treated cells can regulate a number of genes involved in viral processes and pro-inflammatory pathways (Fig. 3c, d). A total of 121 and 249 genes associated with these pathways were annotated as targets of miRNAs enriched in EVs from WNV-infected and IFN-alpha-treated cells, respectively (Additional file 2: Figure S2).

Therefore, bioinformatics analysis of miRNA targets demonstrated that WNV infection and exposure to IFN-alpha increase levels in EVs of miRNAs capable of regulating host genes involved in virus-host interactions, inflammation and innate immune response.

### Levels of specific sncRNAs increase in EVs secreted by WNV-infected cells

Small RNA cargo of EVs is not limited to miRNAs; other classes of host small noncoding RNAs could also be incorporated and transferred by EVs [37]. Therefore, we analysed the levels of small noncoding RNAs in EVs secreted from WNV-infected and IFN-alpha-treated cells.

We found that 41 sncRNAs (Fig. 3e, Additional file 4: Table S3) exhibited increased levels in EVs secreted by WNV infected cells and 30 sncRNAs were increased in EVs from IFN-treated cells (Fig. 3f, Additional file 4: Table S4). Similar to miRNAs, very few sncRNAs had decreased levels in EVs secreted by WNV-infected cells with only two RNAs decreasing significantly by more than 2-fold (Fig. 3e). In EVs secreted by IFN treated cells 14 sncRNAs were significantly decreased. However, the changes in their levels as well as changes in the levels of IFN-induced EV sncRNAs were very moderate and in most cases lower than 2 log2-fold (Fig. 3f). Together with some variation in sncRNA content observed between biological replicates in EVs from IFN treated cells these data suggest that IFN has rather inconsistent and not very profound effect on incorporation of sncRNAs into EVs.

Thereafter, RNASeq data indicate that WNV infection highly increases secretion in EVs of certain sncRNA, whereas treatment with IFN only slightly alters sncRNA profile of EVs.

### Extracellular vesicles secreted by WNV-infected and IFN-alpha-treated cells contain increased levels of mRNAs coding for antiviral proteins

**Fig. 4:**
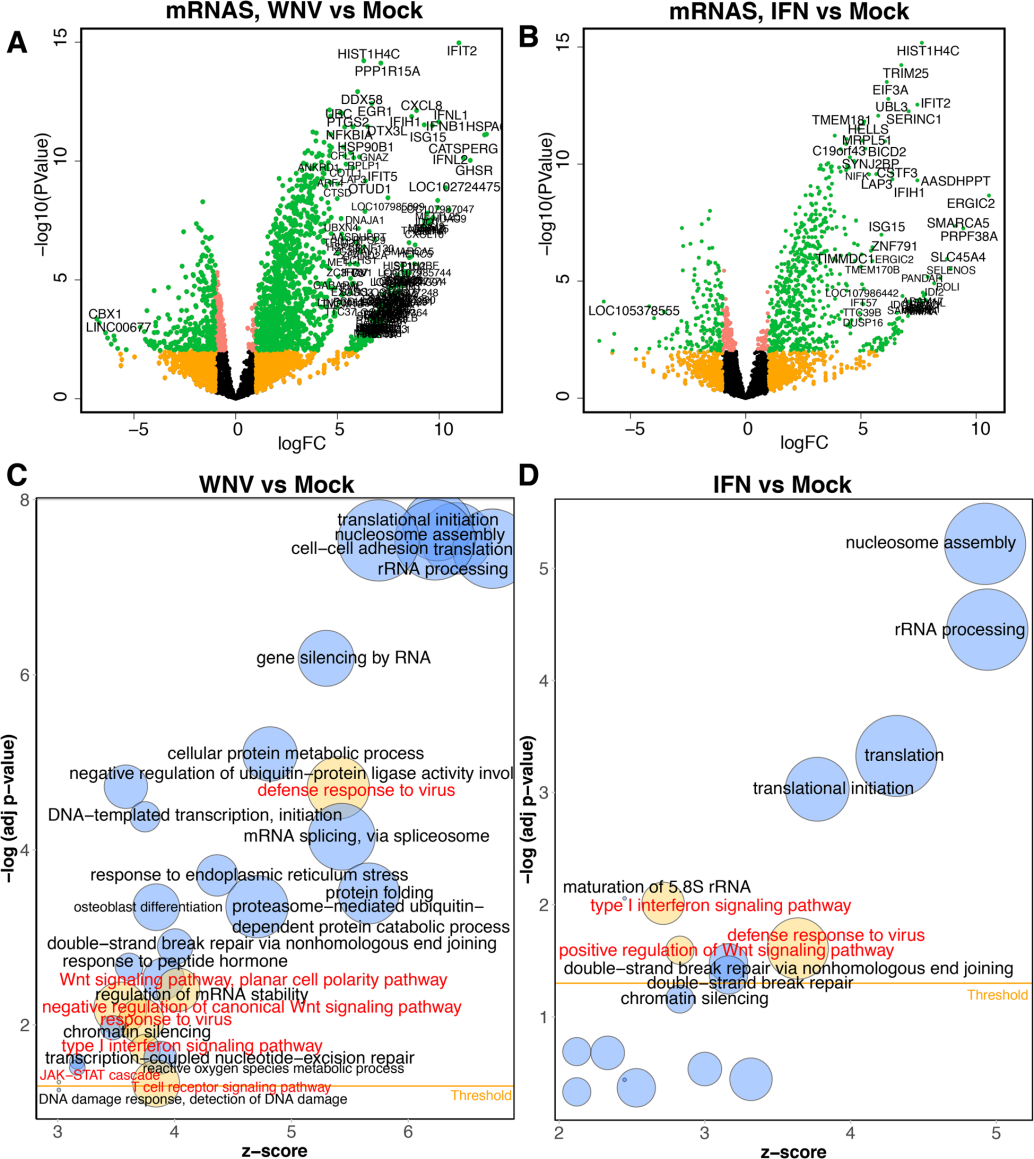
WNV infection and IFN treatment increase secretion of mRNAs associated with antiviral response in EVs. **a** and **b** Volcano plots demonstrate significance and fold change in abundance of mRNAs in EVs secreted from WNV-infected (**a**) and IFN-treated (**b**) A549 cells compared to mock. Red colour highlights mRNAs exhibiting logFC>1, orange indicates mRNAs showing FDR-adjusted *P*-values <0.05 and green indicates mRNAs with logFC>1 and *P*-Value<0.05 (selected for further GO analysis). RNAs with increased abundance are located on the right side ((+) LogFC values) and RNAs with decreased abundance are located on the left side ((-) LogFC values). Gene names are indicated for mRNAs that exhibited the highest change in response to treatment/infection (*P*<0.001, logFC>5). **c** and **d** Bubble diagrams demonstrate GO biological processes significantly associated with mRNAs with increased abundance in EVs secreted by WNV-infected (**c**) and INF-treated (**d**) A549 cells. Size of the bubbles is proportional to the number of increased mRNAs associated with each process, adj p-values are Benjamini-corrected P-values that indicate significance of mRNA enrichment in the process, z-scores indicate direction of change (positive-increase, negative – decrease). Biological processes associated with antiviral response are shown as yellow bubbles with red labels

RNASeq analysis revealed that WNV infection and IFN-alpha treatment also alter secretion of mRNAs in EVs. In EVs from WNV infected cells, 1182 mRNAs had increased level compared to mock and levels of 161 mRNAs were decreased (Fig. 4a, Additional file 4: Table S5). In EVs secreted by IFN-treated cells, 1356 increased and 72 decreased mRNAs were identified (Fig. 4b. Additional file 4: Table S6).

Notably, some increased EV mRNAs code for well-known components of the antiviral response including IFN-β, ISG15, IFIT2, IFNL2, OAS1, NF-kB1, MDA5, etc. (Fig 3a,b, Additional file 4: Table S5, S6). To obtain deeper insights into the biological functions of EV mRNAs affected by WNV infection and IFN-alpha treatment, we identified biological processes associated with EV mRNAs by performing Gene Ontology (GO) enrichment analysis on elevated EV mRNAs. The analysis showed that WNV-induced EV mRNAs were significantly (Benjamini corrected *P*-Value <0.05) enriched in GO categories related to antiviral response (Fig. 4c). In particular, GO analysis identified WNV-induced EV mRNAs to be significantly associated with defence response to virus, Wnt signalling, type I IFN signalling pathway, Jak/STAT cascade and T cell receptor signalling pathway (Fig 4c). IFN-induced EV mRNAs were assigned to the same pathways, processes and networks as WNV-induced EV mRNAs, except for the JAK/STAT signalling and T cell receptor signalling (Fig. 4d). Host mRNAs with decreased levels in either WNV or IFN EVs were not associated with any GO categories related to antiviral response or RNA virus infection (Additional file 4: Table S7, S8). Thus, gene ontology analysis demonstrated that EVs from WNV-infected and IFN-alpha-treated cells carry increased amounts of mRNAs coding for various components of antiviral response pathways.

To identify the exact components of the antiviral pathways encoded by mRNAs selectively incorporated into EVs secreted from WNV-infected cells and their place in virus-induced signalling cascades, the network of regulatory and direct interactions between proteins encoded by these mRNAs was reconstructed. A filter, which retains only genes associated with significantly enriched GO categories related to viral infections and antiviral response identified in GO analysis was then applied to the resulted large network to extract the subnetwork related to antiviral signalling. The filtered network (Additional file 3: Figure S3A) contained well-known pattern-recognition receptors (RIG-I, MDA-5), cytokines (e.g. IFN-b, IL28A/B, IL28, etc), ISGs (ISG15, ISG54, OAS, etc.), positive regulators of type I IFN production (e.g. TRIM25, TRIM56, DDX60, DDX1, etc.), and transcription factors that have specific antiviral functions (e.g. AP-1, IRF-9, IRF-2, IkB). The network also contained proteins with more generic functions (e.g. Hsp90, Actin, G-proteins, etc.), which are shared between different stress response pathways. In general, this network contained the key components of the defence response to RNA viruses.

The same major components of the antiviral response were also identified in the network reconstructed from mRNAs increased in EVs secreted from IFN-alpha-treated cells (Additional file 3: Figure S3B). However, this network was generally smaller (43 vs 78 components) and contained only 29 nods present in the network reconstructed from WNV-induced mRNAs (Additional file 3: Figure S3C). Nonetheless, the mRNAs increased in EVs secreted from IFN-alpha-treated cells coded for well-characterized key components of the antiviral response such as RIG-I, MDA5, IRF1, IRF9, STAT2, ISG15, ISG54 and OAS (Additional file 3: Figure S3B). Moreover, these components contributed to the core part of the antiviral pathways associated with mRNAs increased in EVs secreted from WNV-induced cells as evident by superimposing the networks reconstructed from mRNAs increased in EVs secreted from WNV-infected cells and from IFN-alpha-treated cells (Additional file 3: Figure S3D).

Thus, our analysis indicates that EVs secreted from WNV-infected or IFN-treated cells contain mRNAs coding the critical components of antiviral response.

### Altered incorporation of host RNAs into EVs in response to WNV infection is triggered by combination of IFN-dependent and IFN-independent mechanisms

**Fig. 5:**
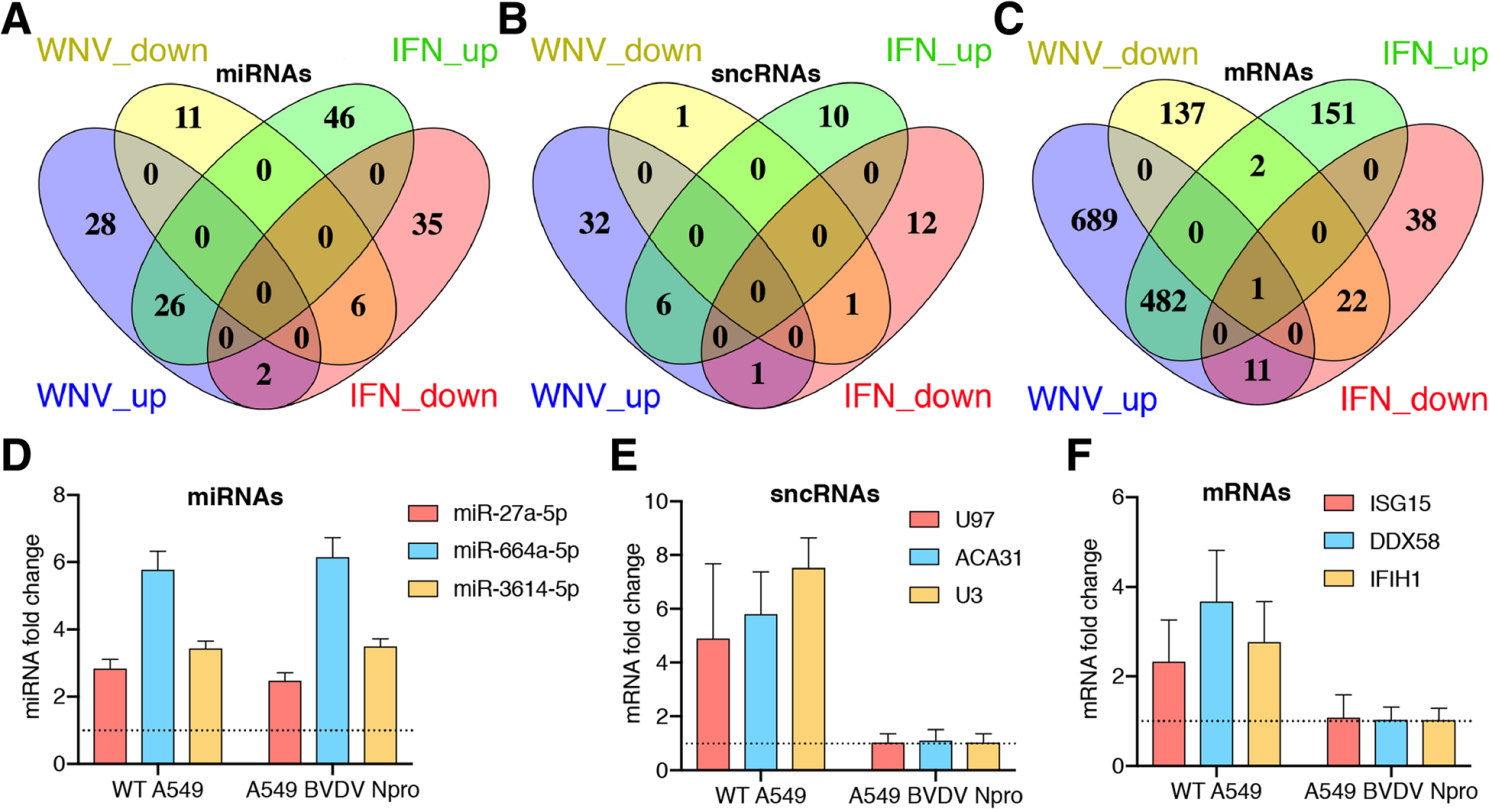
Effect of WNV infection on composition of EVs RNA cargo involves IFN-dependent and IFN-independent mechanisms. Venn diagrams (**a**-**c**) representing common and unique differentially expressed miRNAs (**a**), sncRNAs (**b**) and mRNAs (**c**) identified in EVs secreted by WNV-infected and IFN-treated cells. Bar plots (**d**-**f**) demonstrate the effect of WNV infection on abundance of mRNAs (**d**), sncRNAs (**e**) and mRNAs (**f**) in EVs secreted by IFN-competent (WT A549) and IFN-deficient (A549/BVDV-Npro) cells. RNA abundance was determined by qRT-PCR. Fold change was calculated relative to RNA levels in EVs from mock-treated A549 or A549/BVDV-Npro cells with normalisation to miR-30 (**d**) or SNORD37(**e**, **f**). Values in (**d**-**f**) are the means of 3 biological replicates ±SD. Dash line indicates RNA levels in EVs from mock-treated cells

Our results demonstrated that both, WNV infection and exposure to type I IFN, trigger significant changes in RNA profiles of the secreted EVs. We also found a partial overlap between the sets of mRNAs that code for antiviral proteins with increased incorporation into EVs secreted by WNV infected and IFN alpha treated cells. Taking into account that type I IFNs are the major mediators of the defence response to flaviviruses in vertebrates [75] we hypothesised that IFN signalling can be responsible, at least in part, for the infection-induced changes in composition of EV RNA cargo. If so, there should be a large overlap in the patterns of RNAs that exhibit altered levels in EVs produced after each of the treatments. To elucidate the extend of the similarity between the effect of WNV infection and IFN-alpha treatment on EV RNA profiles we compared the EV RNAs altered in response to WNV infection and to IFN-alpha treatment. The comparison revealed partial overlap in the analysed sets of RNAs. Specifically, 26 common miRNAs (Fig. 5a), 6 common sncRNAs (Fig. 5b) and 482 common mRNAs (Fig. 5c) had elevated levels in EVs after both, WNV infection and IFN-alpha treatment. However, 28 miRNAs, 32 sncRNAs, and 689 mRNAs were uniquely elevated in EVs secreted from WNV-infected cells, while 46 miRNAs, 10 sncRNAs, and 151 mRNAs were uniquely elevated in EVs secreted in response to IFN-alpha treatment (Fig. 5a-c). For EV RNAs with decreased levels, no considerable overlap was found between the responses to IFN-alpha treatment and to WNV infection, as only 6 miRNAs, 1 sncRNA and 22 mRNAs were common (Fig. 5a-c). At the same time, WNV infection led to decreased levels of 11 unique miRNAs, 1 unique sncRNA, and 137 unique mRNAs, while IFN-alpha treatment led to decreased levels of 35 unique miRNAs, 12 unique sncRNAs, and 38 unique mRNAs (Fig. 5a-c).

Therefore, we found that although IFN-alpha treatment and WNV infection commonly increased accumulation of certain coding and noncoding RNAs in EVs, a substantial number of RNAs that showed altered abundance in EVs secreted from WNV-infected cells did not overlap with those induced by IFN-alpha treatment and vice versa. To further investigate the role of IFN signalling in WNV-induced changes of EVs RNA levels we infected A549/BVDV-NPro cells that are deficient in production of type I IFN. These cells stably express N-terminal domain of bovine viral diarrhoea virus (BVDV) protease (BVDV-NPro). BVDV-NPro binds to transcription factors IRF3 and IRF7 that are essential for transcription of IFN genes and induces their ubiquitination with subsequent degradation, rendering the cells incapable of IFN production [76]. We then used qRT-PCR to compare the levels of several miRNAs (Fig. 5d), sncRNAs (Fig. 5e) and mRNAs (Fig. 5f) in EVs secreted by WNV-infected A549/BVDV-NPro cells and wild type (WT) A549 cells. For this analysis host RNAs uniquely increased in EVs from WNV infected cells (miR-27a, U97, ACA31, DDX58) as well as RNAs commonly increased in WNV and IFN EVs (miR-3614, miR-664a, U3, IFIT2, IFIH1) were selected. The results of qRT-PCR demonstrated that WNV infection of WT A549 cells increased levels in EVs of all selected RNAs (Fig. 5e-f) thus confirming the results of RNA-Seq. In IFN-deficient cells the levels of all selected miRNAs increased upon infection, whereas the levels of mRNAs and sncRNAs remained unchanged. Curiously, miR-664a and miR-3614 exhibited elevated levels in EVs produced by both, WNV-infected and IFN-treated cells, according to RNA-Seq data (Fig. 3a,b), while qRT-PCR data indicates that their induction in EVs by infection also occurred in the absence of IFN. This indicates that WNV and IFN can independently increase incorporation of these miRNAs into EVs and that in WNV infection it can occur in IFN-dependent and IFN-independent manner. In contrast to miRNAs, increased incorporation into EVs of selected for qRT-PCR analysis sncRNAs (U97, ACA31, and U3) as well as mRNAs coding for ISG15, DDX58, and IFIH1 appeared to be IFN-dependent as it was not observed in IFN-deficient cells (Fig. 5e,f). However, the results of RNASeq profiling demonstrated that IFN treatment did not lead to increased incorporation into EVs of U97 and ACA31 sncRNAs or DDX58 mRNA and had very modest effect on levels of all sncRNAs. Therefore, IFN treatment is required, but not sufficient to increase incorporation of certain sncRNAs and mRNAs into EVs.

Thus, our results suggest that a complex combination of IFN-dependent and IFN-independent pathways act in WNV-infected cells to increase incorporation into EVs of certain coding and noncoding RNAs in EVs.

### Small RNAs incorporated into EVs secreted by WNV-infected cells have immunostimulatory activity

**Fig. 6:**
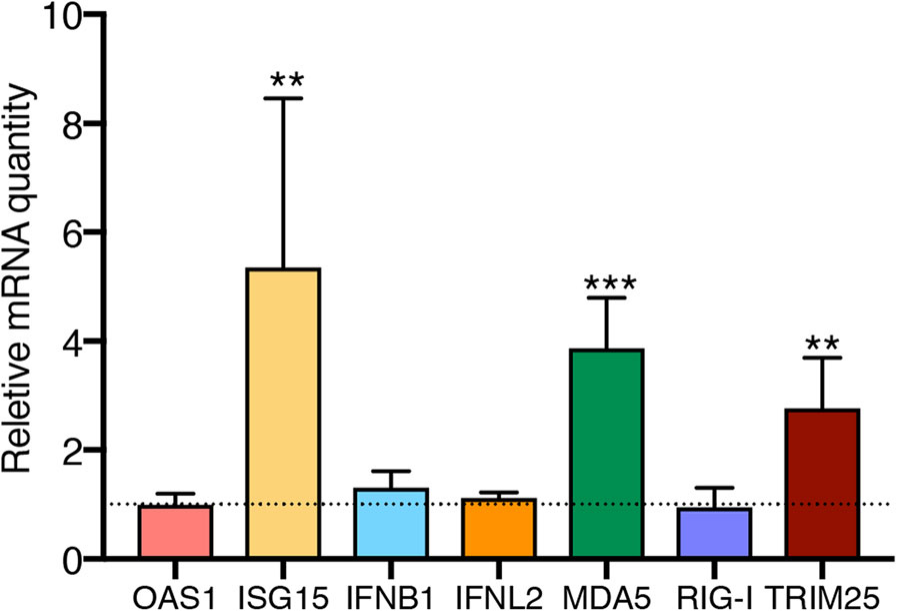
Expression of antiviral genes in A549 cells transfected with small RNA isolated from EVs produced by WNV-infected cells. A549 cells were transfected with small RNA isolated from EVs secreted by WNV-infected or uninfected (mock) cells and gene expression was determined at 24h post transfection by qRT-PCR. Gene expression was calculated by ΔΔCt method relative to the cells transfected with small RNAs from mock EVs and using expression level of housekeeping gene TBP as a reference. Values are the means of 3 biological replicates ±SD. Dash line indicates RNA levels in cells transfected with mock EVs RNA. Statistics analysis was performed using multiple t-tests with Bonferroni correction. ***P<0.001, **P<0.01

To elucidate if RNA from WNV EVs can stimulate innate immune response in acceptor cells, the ability of EV RNA to induce expression of antiviral genes was tested. The enriched fractions of long (>300nt) and small (<300nt) RNAs were isolated from mock and WNV EVs and transfected into A549 cells. At 24h after treatment, cells transfected with long RNAs from EVs showed severe cytotoxic effect and were not suitable for further analysis. Cells transfected with small RNAs displayed normal morphology and were used to determine the expression of selected antiviral genes. Expression of IFNB1, IFNL2, RIG-I, MDA5, TRIM25 and ISG15, which are the well-characterized components of the antiviral response to WNV, was determined by qRT-PCR. Expression of IFNB1, IFNL2, OAS1 and RIG-I showed no difference between cells transfected with small RNA from mock and WNV EVs (Fig. 6). However, expression of MDA5, TRIM25 and ISG15 in cells transfected with small RNAs from WNV EVs was significantly increased compared to those transfected with small RNA from mock EVs (Fig. 6).

These results demonstrate the ability of small RNAs incorporated into WNV EVs to induce expression of several genes involved in innate immune response against RNA viruses, suggesting the immunostimulatory properties of small RNAs incorporated into EVs secreted by WNV-infected cells.

## Discussion

Extracellular vesicles are small membrane-covered spherical vesicles that transfer their RNA and protein cargo between the cells of the multicellular organism [21]. Several studies have previously demonstrated that EVs are involved in virus-host interactions particularly by transferring infectious viral RNA [43, 44] and virus virulence factors [77–79], antiviral proteins APOBEC3G [46], antiviral miRNAs [80–82] and mRNAs encoding for antiviral proteins [83]. In flavivirus infection, exosomes secreted by DENV-infected cells have been shown to contain proteins, miRNAs and mRNAs associated with antiviral response and transfer antiviral activity to the recipient cells [17, 47]. Here we have shown that infection with WNV also induces significant changes in secretion of coding and noncoding RNAs in EVs and these RNAs are associated with antiviral response.

A number of studies have reported differential incorporation of miRNAs into EVs and the ability of EV miRNAs to alter expression of their targets in recipient cells, modulating various regulatory pathways including antiviral response (reviewed in [39]). In addition, miRNAs are well-known regulators of host response to flaviviruses, including WNV [84–88]. The ability of extracellular vesicles to transfer miRNAs that modulate viral infection was first described in HIV infection. Exosomes produced by primary alveolar macrophages infected with HIV were shown to contribute to chronic immune activation in HIV-infection as they contain viral microRNAs vmiR88 and vmiR99 responsible for stimulation of TNFα release in human macrophages [45]. In addition, expression of HIV Nef protein in macrophage-like cells was shown to recruit 47 host miRNAs into exosomes [77]. TLR3-activated macrophages have been shown to release EVs that contain members of miR-29 family and inhibitory effect of EV miR-29 on replication oh HCV in hepatocytes has been demonstrated [80]. Considering the profound role of exosomal miRNAs in modulation of host response to viruses, we first analyzed levels of miRNAs in EVs secreted by WNV-infected cells.

To determine the levels of miRNAs in EVs we employed next generation sequencing of RNA (RNASeq). This was conducted in two biological replicates because, considering that genetically homogenous cell line grown under standardized conditions was used as an experimental model, we did not expect a high degree of variability between independent experiments. This assumption was confirmed by multidimensional scaling analysis, which demonstrated that all replicates except sncRNAs from IFN-treated cells had high degree of similarity as indicated by close positioning of replicates in Fig. 2a-c. In addition, the majority of RNAs with expression levels higher than 5 cpm had very low biological coefficients of variations (Additional file 1: Figure S1). We also employed generalised linear model with quasi-likelihood F-test as a method for statistical analysis, which is known to produce reliable results with small numbers of biological replicates [89]. Although we can’t exclude that limitation may apply to interpretation of RNA-Seq results from experiment with small number of replicates, particularly for low abundance transcripts, computational data exploration (Fig. 2, Additional file 1: Figure S1) have thus demonstrated that for the vast majority of the transcripts we have likely obtained confident expression data.

We identified 56 miRNAs that were enriched in EVs secreted from WNV-infected cells. Five of these miRNAs (miR-148a, miR-494, miR-708, miR-483 and miR-29b) were previously found to be significantly enriched in exosomes from HIV Nef-expressing macrophages [77]. This indicates that incorporation of these miRNAs into EVs may represent a general and conservative mechanism of antiviral response and suggests their importance in EV-mediated signalling in viral infection. Notably, IFN-alpha treatment also resulted in increased levels of 72 miRNAs in EVs.

Interestingly, more miRNAs were significantly enriched in EVs secreted from WNV-infected and IFN-alpha-treated cells than miRNAs that were decreased. In addition, miRNAs enriched in EVs from WNV-infected and IFN-alpha-treated cells exhibited much higher difference in abundance compared to EVs secreted from Mock cells, than miRNAs that were decreased. This observation was consistent with data on EVs from HIV Nef-expressing cells, in which levels of decreased miRNAs were changed by not more than 0.6-fold [77]. This suggest that cellular response to infection more likely relies on increased incorporation of miRNAs into EVs rather than on their retention in cells and supports the signalling model in which infection leads to the appearance of the new signal rather than decrease of some basal stimuli in EVs.

Some of the enriched EV miRNAs identified in our study were previously reported to regulate genes involved in antiviral response. Particularly, miR-29b is known to target tumour necrosis factor-induced protein 3 in response to JEV infection and is thought to be required for JEV-induced microglia activation [90]. This miRNA has also been found responsible for exosomal transfer from activated macrophages to hepatocytes of antiviral activity against HCV [80]. Human miR-27a has an inhibitory effect on HCV [91], EV71 [92] and adenovirus [93], and regulates antiviral response via TRIM27 [94]. MicroRNA miR-34c was shown to induce apoptosis in hepatitis B infected cells [95]. MicroRNA miR-26a suppresses replication of porcine reproductive syndrome virus [96] and is also down-regulated by influenza A virus to reduce production of type I IFN [97].

To obtain a complete picture of relationships between miRNA enriched in EVs, and their targets, we reconstructed the miRNA-target networks based on the list of experimentally validated targets for enriched miRNAs in EVs produced by WNV-infection and IFN-alpha-treatment. The resulted networks contained numerous interactions between miRNAs enriched in WNV infection-induced EVs and the host genes involved in multiple viral processes. This analysis demonstrated strong association between regulation of virus-host interactions and miRNAs miR-26a, miR-29, miR-3929, miR-215, miR-520a, miR-24. These results together with currently known antiviral role of above-mentioned infection-induced EV miRNAs are intriguing as they suggest that EVs produced upon WNV infection may potentially transfer the antiviral or pro-inflammatory signal.

The wide range of targets associated with viral infection was also identified for miRNAs enriched in EVs secreted from IFN-alpha-treated cells, suggesting that previously described role of EVs in transduction of IFN-induced pro-inflammatory signal [14, 46, 69] may not be limited to the transfer of antiviral proteins.

In addition, we have found that WNV infection increased levels in EVs of a number of host small noncoding RNA, primarily small nucleolar RNAs (snoRNAs). Similar to miRNAs, the majority of sncRNAs were enriched rather than depleted in EVs. This suggests that they are actively sorted into EVs and could potentially contribute to the transfer of antiviral signal. However, their potential role in WNV infection is puzzling as sncRNAs are less well-studied than miRNAs and very little is known about their role in virus-host interactions. One study demonstrated that a broad range of snoRNAs can activate antiviral enzyme PKR [98]. In addition, processing of snoRNAs into miRNAs [99] and piRNAs [100] has also been reported. Further experiments are required to determine the biological significance of the altered levels of sncRNAs in EVs and their role in virus-host interactions.

Interestingly, we found that transfection of small RNAs isolated from EVs secreted by WNV infected cells results in increased expression of genes encoding for MDA5 and TRIM25, which are involved in viral RNA sensing [101, 102] as well as ISG15, which is a known antiviral effector protein [103]. This indicates that small RNAs incorporated into EVs can have immunostimnulatory effect. However, they appear not to induce complete antiviral response pathway in target cells as we could not detect activation of other genes essential for virus elimination including INFs and OAS. We hypothesise that delivery of small RNAs by EVs secreted from infected cells may “precondition” neighbouring cells for faster antiviral response without activation of the antiviral response to its full capacity as the later can potentially result in cell death. However, this hypothesis as well as the identity of small RNAs responsible for the observed effect requires extensive further validation.

We also demonstrated that WNV infection increased abundance of mRNAs encoding for the components of antiviral pathways in EVs. EVs from both, WNV-infected and IFN-treated cells contained increased levels of mRNAs encoding for pattern recognition receptors, transcription factors regulating inflammation and antiviral signalling, cytokines, their receptors and effectors of antiviral pathway. One of this mRNA, encoding for DDX58, was previously reported to incorporate into EVs secreted by DENV-infected dendritic cells [47]. In our study DDX58 mRNA was found to be increased in EVs from WNV-infected and IFN-treated cells and was also confirmed in WNV EVs by qRT-PCR. Some EV-transferred mRNAs have been previously shown to be translated in recipient cells altering their signalling pathways [23], however the role of EV-incorporated host mRNAs in viral infection remains elusive. The role of exosomal mRNA delivery in regulation of antiviral response has been recently suggested, when exosomes with anti-HIV activity were isolated from human semen and demonstrated to contain mRNAs encoding for antiviral factors, however this assumption was primarily based on indirect evidences [83]. Unfortunately, due to cytotoxicity we were unable to test if mRNAs from EVs secreted by WNV-infected cells can alter the antiviral state of the target cells and thus cannot conclusively claim the role of EV-derived mRNAs in infection. We can, however, speculate that mRNAs secreted in EVs from WNV-infected cells may become translated upon delivery to the target cells and modulate their antiviral state. Alternatively, EVs can be employed to remove excess mRNAs transcribed from the antiviral genes in infected cells or products of their incomplete degradation to prevent overload of the translation machinery or accumulation of waste products within the cells. Removal of cellular waste was the first function suggested for EVs [19] and it is possible that mRNAs accumulated in EVs secreted by infected cells reflect the role of EVs related to the homeostasis of maternal cells and not to altering the pathways of target cells. Therefore, further studies are required to dissect functions of EVs in transport of mRNAs and their role in viral infection.

Our study has also demonstrated that WNV infection and treatment with IFN induce common and unique changes in RNA profiles of EVs. In addition, we found that IFN-alpha treatment increased levels in EVs of larger number of miRNAs that target wider network of genes associated with viral processes than WNV infection. However, three miRNAs that we tested for incorporation into EVs in IFN-deficient cells, responded to infection in the same way as in WT cells. These observations can be explained by suggesting that IFN-alpha can induce incorporation of miRNAs into EVs, but there is a redundancy in the pathways that control EV levels of miRNAs so they can also be activated in response to infection in IFN-independent manner. The more profound effect of IFN on miRNAs levels in EVs compared to WNV infection can therefore be explained by possible suppression of antiviral pathways in infected cells by viral proteins or noncoding RNA [3, 104, 105].

Intriguingly none of tested 3 sncRNAs and 3 mRNAs, including those that were uniquely increased in EVs from WNV infected and not from IFN-treated cells, exhibited increased levels upon WNV infection of IFN-deficient cells. This indicates that secretion in EVs of these RNAs requires activation of IFN-inducible pathways. However, IFN-treatment alone is not always sufficient to trigger incorporation of mRNAs and scnRNAs into EVs as increased levels of some infection-induced RNAs have not been observed in EVs from IFN-treated cells. Thus their secretion in EVs requires activation of the infection-induced IFN-independent pathway in addition to IFN-induced pathway.

Therefore, our data indicate that levels of host RNAs in EVs secreted by the infected cells depend on the complex combination of IFN-dependent and IFN-independent pathways that have some degree of redundancy, particularly in the control of EV-incorporated miRNA levels. The demonstrated here ability of small RNAs from EVs to induce expression of certain antiviral factors in transfected cells suggest their likely biological importance in flavivirus-host interactions. However, further studies are required to identify which specific small RNAs are involved in the induction of antiviral response as well as whether EV-incorporated mRNAs can also contribute to antiviral signalling.

## Conclusions

We have shown that WNV infection and treatment with IFN-alpha alters the levels of various host RNAs in EVs. Host miRNAs that exhibit increased levels in EVs secreted from WNV-infected and IFN-treated cells are known regulators of viral processes and antiviral response while EV-incorporated mRNAs code for known antiviral proteins. Increase in EVs levels of host RNAs is controlled by a combination of partially interconnected IFN-dependent and IFN-independent pathways. Our study provides the first comprehensive profiling of host RNAs incorporated into EVs secreted by flavivirus-infected cells, demonstrates the role of type I IFN-dependent and independent pathways in regulating levels of these host RNAs in EVs during infection, and predicts functional associations of these host RNAs with antiviral processes in acceptor cells.

## Methods

### Cell culture

Human lung carcinoma cells (A549) [ATCC CCL-185] and A549/BVDV-NPro cells [76] were cultured in F12K medium supplemented with 10% heat-inactivated FBS. Green monkey kidney cells Vero and hamster kidney cells BHK-21 were maintained in Dulbecco’s Modified Eagle Medium supplemented with 5% heat-inactivated FBS. All cell culture media contained 100 units penicillin ml^−1^, 100 μg streptomycin ml^−1^, 0.29 mg l-glutamine ml^−1^ (Gibco) and 1 mM sodium pyruvate. All cells were maintained at 37 °C in CO_2_ incubator. FBS was from SAFC Biosciences (Australia), other cell culture reagents were from Gibco, Life Technologies Inc. (USA).

### Virus infection and IFN-alpha treatment

West Nile virus Kunjin strain was obtained from the full-length cDNA clone as described previously [106]. Virus was then amplified in Vero cells and titres were determined by plaque-assay in BHK-21 cells: serial dilutions of culture fluids were prepared, and 200 μl of each dilution was used to infect BHK-21 cells grown in 6-well plates, followed by incubation for 2 h and overlaying with DMEM supplemented with 0.5% low-melting-point agarose (Rio-Rad, USA) and 2% FBS. At 72 h after infection, cells were fixed with 4% formaldehyde in PBS and stained with 0.2% crystal violet solution. Plaques were then counted, and titres were calculated based on the dilution factor and infected volume.

All infections were performed at multiplicity of infection (MOI) = 1 by incubation of cells with 45 μl of inoculum per cm^2^ of growth area for 2h at 37^o^C. After inoculum was removed, the cells were washed twice with PBS, and fresh medium was added. Human interferon alpha 2a (Roche, USA) was added to cells at final concentration of 300u/ml for 24h. Infected and IFN-treated cells were maintained in F12K medium supplemented with 2% FBS.

### Isolation of extracellular vesicles (EVs)

A549 (infected, IFN-treated and mock) cells grown in monolayers in T175 flasks at ~80% confluence were washed 3 times with PBS and then 20ml F12K medium supplemented with 2% exosomes free FBS (Gibco, USA) were added to the monolayer. Cells were allowed to secrete EVs for 24h, then culture fluids were collected. Culture fluids from 2xT175 flasks (~4x10^7^ cells) were combined and subjected to centrifugation at 5000xg for 1h at 4^o^C to remove cell debris and apoptotic bodies. To inactivate and remove contaminating virus particles prior to exosome extraction, 1u/ml RNaseA (Sigma, USA) was added to the conditioned media followed by heating at 56**°**C for 1 hour and centrifugation for 15 min at 3000 x g, 4^o^C. EVs were then isolated from conditioned media using ExoQuick-TC (Systems Bioscience, Mountain View, CA) according to the manufacturer’s protocol.

### Western Blotting

Pelleted EVs were re-suspended in RIPA buffer (Sigma, USA) supplemented with Complete Proteinase Inhibitor Cocktail (Roche, Switzerland) and protein concentration in lysates was determined using BCA Protein Assay (Pierce, USA). Proteins (25 ug/sample) were denatured by heating for 5 min at 95^o^C in Bolt LDS Sample Buffer (Thermo Scientific, USA) supplemented with 1x Reducing Agent (Thermo Scientific, USA) and then separated in 4-12% Bolt Bis-Tris Plus Gel (Thermo Scientific, USA). Electrophoresis was performed for 45 min in Bolt MES Running buffer (Thermo Scientific, USA) at 200V. Proteins were electroblotted onto nitrocellulose membrane (Millipore, USA) for 1h at 400mA in Tris-Gly-SDS buffer with 20% methanol using Mini Trans-Blot Cell (BioRad, USA). Membrane was blocked with 3% non-fat dry milk (NFDM) in TBS for 30 min and then incubated O/N at 4oC with antibodies specific to CD9 (#13174, Cell Signaling Technologies, USA) diluted 1:500, CD63 (#310628D, Thermo Scientific, USA) diluted 1:250, or Hsp70 (sc-24, Santa Cruz Biotech., USA) diluted 1:100. All antibodies were diluted in TBST. Membranes were then washed 4 x 5min with TBST and incubated for 1h at room temperature with anti-rabbit or anti-mouse HRP-conjugated antibody (Cell Signaling Technologies, USA) diluted 1:5000 in 3% NFDM in TBS. After 4 washes with TBST, signal was developed using ECL Plus Reagent (Thermo Scientific, USA) and visualized on AI600 imager (GE Healthcare, USA).

### Electron microscopy

Sample was absorbed onto carbon-coated copper grid for 2 mins at room temperature. Samples were rinsed with PBS, then washed 4x5min in PBS and 1x5min with dH2O. Samples were contrasted with 1.8% methyl cellulose/ 0.4% uranyl acetate on ice, 5 mins then looped out and dried. The sections were viewed on a JOEL 1010 transmission electron microscope, and images were captured using a MegaView III CCD camera (Soft Imaging Systems, USA).

### Fluorescent labelling of EV RNA and confocal microscopy

Fluorescent labelling of EV RNA was performed by incubating EVs (1 μg in 160 μl) with 1.6 μl of 1 μM SYTO RNASelect reagent (Thermo Scientific, USA) according to manufacturer’s instructions. Unincorporated dye was removed using MW3000 exosome purification spin columns (Thermo Scientific, USA). Suspension of labelled EVs (100 ng) was added to culture media of A549 cells growing on cover slips in wells of 24-well plate and incubated for 16h. Culture media was then removed, cells were washed twice with cold PBS and fixed for 15 min with cold 4% PFA in PBS followed by two washes with cold PBS. Cover slips were mounted on glass sides and nuclei were counter-stained with DAPI using ProLong Diamond Antifade Mountant (Thermo Scientific, USA). Fluorescent signal in the cells was visualized on ZEISS LSM 510 META Confocal Microscope.

### RNA isolation

Enriched small and long RNA fractions from EVs were isolated using mirVana miRNA Isolation Kit (Ambion, USA). Viral RNA from EVs and culture fluids was isolated using NucleoSpin RNA Virus Kit (Macherey-Nagel, Germany). Total RNA for qRT-PCR was isolated from EVs pellets or cultured A549 cells using TRI Reagent (Sigma-Aldrich, USA). Concentration of RNA was determined on Nanodrop ND-1000 (Thermo Scientific, USA). The ratios of OD_260_/OD_280_ and OD_260_/OD_230_ were used to assess the purity of RNA preparations. If prepared for RNA-Seq analysis, RNA was analysed and quantified on 2100 Bioanalyzer Instrument (Agilent Technologies, USA).

### RNA transfection

A549 cells (10^6^) were transfected in suspension with 500ng of small or long RNA isolated from EVs using 12.5 μl of Lipofectamine 2000 as described previously [84]. Total RNA was isolated from transfected cells at 24h after transfection.

### RT-PCR

RNA (5ul) was subjected to RT-PCR using SSIII One-Step RT-PCR Kit with Platinum Taq (Invitrogen USA) under the following conditions: 60^o^C – 15min, 95^o^C – 5min, followed by 35 cycles of 95^o^C – 15″, 60oC – 30″, 68oC – 15″ and final extension at 68^o^C for 5 min with 10pmole of each primer (Additional file 4: Table S9).

### Quantitative RT-PCR

Total cellular RNA (1μg) was used for cDNA synthesis with qScript cDNA SuperMix (Quantabio, USA) according to manufacturer’s instruction. cDNA was diluted 1:10 and 3 μl of cDNA solution were used as template for qRT-PCR using SYBR Green PCR Master Mix (Applied Biosystems, USA). PCR was performed in 20 μl of reaction mix containing 10 pmoles of forward and reverse PCR primers for each gene. Reactions were performed under the following cycling conditions: 95°C for 5 min and 40 cycles of 95°C for 15 s and 60°C for 1 min, followed by melting-curve analysis.

For the analysis of EV miRNAs total EV RNA was used for cDNA synthesis using stem loop RT primers [107] and TaqMan MicroRNA Reverse Transcription Kit (Thermo Fisher Scientific, USA). Each RNA sample was subjected to the reaction with a pool of RT primers for all analysed miRNAs. Each 40ul reaction contained 100ng RNA, 1pmole of each primer 400u MultiScribe Reverse Transcriptase, 10u RNase Inhibitor, 2mM dNTPs and 1x RT buffer. Reaction was incubated at 16^o^C for 30 min, followed by 30 min at 42^o^C and 5 min at 85^o^C.

For the analysis of EV mRNAs and sncRNAs total ER RNA (100ng) was used as a template for cDNA synthesis using qScript cDNA SuperMix (Quantabio, USA). Reaction was performed according to the manufacturer’s instructions.

To enable amplification of the multiple targets using small amount of starting RNA material, cDNA for qRT-PCR of EVs miRNAs, mRNAs and sncRNAs was preamplified according to the published protocol [108]. Each cDNA sample (5ul) was used as a template for PCR with a pool of all relevant primers (0.5 pmole each) and Taq Master Mix (NEB, USA) in 50ul volume. Preamplification was performed by incubation at 95^o^C for 10 min followed by 14 cycles of 15 sec at 95^o^C and 4 min at 60^o^C. Enzyme was inactivated by heating at 99^o^C for 10min, cDNA was diluted 1:4 with water and 5ul (miRNAs) or 3ul (mRNAs, sncRNAs) of diluted DNA were used as a template for qRT-PCR.

Quantitative RT-PCR was conducted in 20 ul (miRNAs) or 10ul (mRNAs, sncRNAs) of reaction mix containing 10 pmole of each primer and QuantaNova SYBR Green PCR Master Mix (Quiagen, USA). Reactions were performed under the following cycling conditions: 95°C for 2 min and 40 cycles of 95°C for 5 s and 60°C for 20sec, followed by melting-curve analysis.

All qRT-PCR reactions were performed using QuantStudio 6 Flex Real Time PCR Instrument (Applied Biosystems, USA). Gene expression analysis was performed by the ΔΔ*C*_*T*_ method using QuantStudio software (Applied Biosystems, USA). Gene expression in RNA transfection experiments was normalized to the TATA-binding protein (TBP) mRNA level and compared to samples of Mock EVs RNA transfection. In EVs profiling experiments data were normalised to miR-32 (miRNA) or SNORD37 (mRNA, sncRNA). Reference genes were selected based on their uniform expression between the samples according to RNAseq data, snoRNA was used as a reference for mRNA analysis as mRNAs of typical housekeeping genes (GAPDH, TBP, etc) was not detected in EVs at considerable levels.

For each experiment, RNA from 3 biological replicates was used and PCR amplification of each cDNA sample was done in technical triplicate. Negative controls were included for each set of primers.

### Next generation sequencing

For RNA-Seq profiling, RNA was isolated from EVs, DNase treated using RQ DNaseI (Promega, USA) and purified by phenol:chlorophorm extraction and ethanol precipitation. cDNA libraries from small RNAs were prepared using TrueSeq Small RNA Library Preparation Kit (Illumina Inc., USA), mRNA libraries were prepared using a TruSeq RNA Library Prep Kit (v2) (Illumina Inc., USA). cDNA libraries were sequenced (50 bp single end reads) using the Illumina MiSeq Sequencer (Illumina Inc.). The primary sequence data was generated using the Illumina CASAVA1.8.2 pipeline and stored in FASTQ format. Library preparation and sequencing was performed by Australian Genome Research Facility (Melbourne, Australia).

Illumina adapters were trimmed, PCR primers, contaminants, low quality reads and reads under 15 nts in length were removed from primary data using Trimmomatic v0.36 with settings ILLUMINACLIP: TruSeq3-SE:2:30:10 LEADING:3 TRAILING:3 SLIDINGWINDOW:4:15 MINLEN:15. Quality of remaining reads was assessed using FastQC v0.11.5 (http://www.bioinformatics.babraham.ac.uk/projects/fastqc/). Reads were then mapped to human genome assembly hg38 using STAR v2.5.2 [109] allowing 1 mismatch and stored in BAM files. The counts of reads mapping to each known gene were determined at transcript level using the HTSeq v0.7.0 [110] using union model and counting to “exon” feature. For counting the RNA transcripts and small noncoding RNAs from EVs, gene feature format (GFF) files were obtained from UCSC genome browser (assembly GRCh38/hg38, Dec. 2013). GFF files with coordinates of miRNAs were obtained from miRBase (release 21). The read counts were used to determine gene expression and identify differentially expressed genes (DEGs) using R package ‘edgeR’ v3.10.5 [111]. Low count transcripts (<1 rpm (reds per million) for miRNAs and <0.5 rpm form mRNAs) were omitted from the analysis and remaining counts were normalized to the library size using TMM normalization method of edgeR. The GLM model was used to perform differential expression comparison between the groups by quasi likelihood F-test. Differentially expressed genes were considered significant if the FDR value was < 0.05. Sequencing experiments were performed with two biological replicates.

Raw data of next generation sequencing are available in GEO database (GSE114008).

### Identification and Analysis of miRNA targets

Visualization and functional annotation of miRNA-mRNA interactions was performed using miRNet software [112]. Lists of experimentally validated mRNA-miRNA interactions were extracted from miRNet database, then mRNAs targeted by<2 miRNAs and miRNAs targeting <2 mRNAs were removed from the list by applying the filter with “degree cut-off” =1 in miRNet software. The resulted interactions were used to reconstruct the network miRNA-target networks. The networks were subjected to functional exploration in which enriched KEGG pathways associated with the network components were identified (A, B, E, F) and subnetworks associated with KEGG pathways related to virus-host interactions were extracted (C, D, G-I).

### Functional Classification of differentially expressed genes and network modelling

GO analysis and functional classification of differential WNV EV RNAs was performed using DAVID v6.8 [113]. Lists of differentially expressed genes were submitted to the tool and GO enrichment analysis was performed with following settings: GO category – GOTERM_BP_DIRECT, Thresholds: counts=5, EASE=0.1, correction for multiple comparisons was Benjamini. Output table from DAVID was combined with gene expression levels using R package GOplot [114], which was then used to perform gene set analysis and plot the result with overlap reduction value set to 0.75. Network reconstruction was performed using GeneGo MetaCore Softaware (Thomson Reuters, USA). List of differentially incorporated mRNAs with log2-fold change values and P-values was submitted to MetaCore and network reconstruction was performed using “direct interactions” algorithm, which assigns network edges to the experimentally validated interactions between the genes present in manually curated MetaCore database. Then MetaCore filter was applied to the resulted large network, which allows selection of GeneGo terms from the list of terms significantly associated with the genes present in the network. Terms related to antiviral response and sensing of viral RNA were selected from the list and genes that are not assigned to these terms were hidden from the network.

### Statistical analysis

Statistical testing for differentially expressed genes in RNA-Seq experiments was performed using edgeR. Analysis of qRT-PCR was performed by multiple t-tests with Bonferroni correction using Prism v8.0 (GraphPad, USA).

## Additional files

Additional file 1: **Figure S1.** Characterization of RNASeq datasets. Box plots represent abundance distribution for all RNAs in datasets before and after normalisation and BCV plots show variations between the biological replicates after normalisation for EV miRNAs (A), sncRNAs (B) and mRNAs (C). (PDF 296 kb)

Additional file 2: **Figure S2.** Targets of miRNAs differentially incorporated into EVs from WNV-infected (A) and IFN-treated (B) A549 cells associated with virus-related pathways. To obtain the networks lists of experimentally validated mRNA-miRNA interactions for each miRNA dataset were extracted from miRNet database, then mRNAs targeted by<2 miRNAs and miRNAs targeting <2 mRNAs were removed from the list by applying the filter with “degree cut-off” =1 in miRNet software. The resulted interactions were used to reconstruct the networks, which then were subjected to functional exploration in which enriched KEGG pathways associated with the network components were identified and subnetworks associated with KEGG pathways related to virus-host interactions were extracted. (TIF 6758 kb)

Additional file 3: **Figure S3.** Comparison of the antiviral pathway-associated networks reconstructed from mRNAs with increased abundance in EVs from WNV-infected or IFN-alpha-treated cells. (A, B) Simulated networks of the components of antiviral pathways encoded by mRNAs increased in EVs secreted from WNV-infected (A) or IFN-alpha-treated (B) cells. (C) Numbers of unique and common network nods associated with antiviral response that are enriched in EVs secreted from WNV-infected and IFN-alpha-treated cells. (D) Results of superimposing (B) to (A) indicating which of WNV-induced EV mRNAs and antiviral pathways overlap with those induced by IFN-alpha treatment. Red (induction) and blue (repression) circles near the nods indicate the changes in mRNA level in WNV EVs (A) or IFN-alpha EVs (B, D) with the intensity of the colouring indicating the level of change. Red edges indicate inhibitory and green edges indicate stimulatory interactions between network components, grey edges indicate that the effect of interaction is not known; symbols of the nods indicate functional category of the network component (https://portal.genego.com/legends/MetaCoreQuickReferenceGuide.pdf). To reconstruct the networks, list of the differentially incorporated mRNAs with log2-fold change values and P-values were submitted to MetaCore software and network reconstruction was performed using “direct interactions” algorithm, which assigns network edges to the experimentally validated interactions between the genes present in manually curated MetaCore database. Then MetaCore filters were applied to the resulted large networks, which allows selection of GeneGo terms from the lists of terms significantly associated with the genes present in the network. Terms related to antiviral response and sensing of viral RNA were selected from the list and genes that are not assigned to these terms were hidden from the network. The resulted simplified networks, which only contains components directly associated with antiviral activity, are shown in the figure. (TIF 3701 kb)

Additional file 4: **Table S1.** EV miRNAs significantly (FDR<0.05, logFC>1) altered in response to WNV infection comparing to mock EVs. **Table S2.** EV miRNAs significantly (FDR<0.05, logFC>1) altered in response to IFN-alpha treatment comparing to mock EVs. **Table S3.** EV sncRNAs significantly (FDR<0.05, logFC>1) altered in response to WNV infection comparing to mock EVs. **Table S4.** EV sncRNAs significantly (FDR<0.05, logFC>1) altered in response to IFN-alpha treatment comparing to mock EVs. **Table S5.** EV mRNAs significantly (FDR<0.05, logFC>1) altered in response to WNV infection comparing to mock EVs. **Table S6.** EV mRNAs significantly (FDR<0.05, logFC>1) altered in response to IFN-alpha treatment comparing to mock EVs. **Table S7.** GO terms associated with mRNAs exhibiting decreased levels in EVs secreted by WNV infected A549 cells. **Table S8.** GO terms associated with mRNAs exhibiting decreased levels in EVs secreted by IFN-alpha treated A549 cell. **Table S9.** PCR primers used in the study. (XLSX 349 kb)

## Data Availability

The dataset supporting the conclusions of this article are available in the Cene Expression Omnibus (GEO) repository, accession number GSE114008.

## Abbreviations

EVs: Extracellular vesicles
IFN: Interferon
miRNA: microRNA
MVs: Microvesicles
sncRNA: small noncoding RNA
snoRNA: small nucleolar RNA
WNV: West Nile virus

## References

[1] Brinton MA (2013). Replication cycle and molecular biology of the West Nile virus. Viruses..

[2] Roby JA, Funk A, Khromykh AA, Shipy I (2012). Flavivirus replication and assembly. Molecular virology and control of flaviviruses.

[3] Slonchak A, Khromykh AA (2018). Subgenomic flaviviral RNAs: What do we know after the first decade of research. Antiviral Res.

[4] Clarke BD, Roby J, Slonchak A, Khromykh AA (2015). Functional non-coding RNAs derived from the flavivirus 3’ untranslated region. Virus Res..

[5] Chancey C, Grinev A, Volkova E, Rios M (2015). The global ecology and epidemiology of West Nile virus. Biomed Res Int..

[6] Lim SM, Koraka P, Osterhaus ADME, Martina BEE (2011). West Nile virus: Immunity and pathogenesis. Viruses..

[7] Brinton MA (2002). The molecular biology of West Nile Virus: a new invader of the western hemisphere. Annu Rev Microbiol..

[8] Hughes JM, Wilson ME, Sejvar JJ (2007). The long-term outcomes of human West Nile virus infection. Clin Infect Dis..

[9] Muñoz-Jordán JL, Fredericksen BL (2010). How flaviviruses activate and suppress the interferon response. Viruses..

[10] Nazmi A, Dutta K, Hazra B, Basu A (2014). Role of pattern recognition receptors in flavivirus infections. Virus Res..

[11] Takeuchi O, Akira S (2009). Innate immunity to virus infection. ImmunolRev..

[12] Randall RE, Goodbourn S (2008). Interferons and viruses: An interplay between induction, signalling, antiviral responses and virus countermeasures. J Gen Virol..

[13] Assil S, Webster B, Dreux M (2015). Regulation of the host antiviral state by intercellular communications. Viruses..

[14] Cossetti C, Iraci N, Mercer TR, Leonardi T, Alpi E, Drago D (2014). Extracellular vesicles from neural stem cells transfer IFN-g via Ifngr1to activate Stat1 signaling in target cells. Molcel..

[15] Zhou W, Woodson M (2018). Neupane B, Bai F.

[16] Felli C, Vincentini O, Silano M, Masotti A. HIV-1 Nef signaling in intestinal mucosa epithelium suggests the existence of an active inter-kingdom crosstalk mediated by exosomes. Front Microbiol. 2017;8 JUN:1–8. 10.3389/fmicb.2017.01022 PMC5462933 28642743

[17] Zhu X, He Z, Yuan J, Wen W, Huang X, Hu Y (2015). IFITM3-containing exosome as a novel mediator for anti-viral response in dengue virus infection. Cell Microbiol..

[18] Théry C, Zitvogel L, Amigorena S (2002). Exosomes: composition, biogenesis and function. Nat Rev Immunol..

[19] Johnstone RM, Adam M, Hammond JR, Orr L, Turbide C (1987). Vesicle formation during reticulocyte maturation. Association of plasma membrane activities with released vesicles (exosomes). J Biol Chem..

[20] Hessvik NP, Llorente A (2018). Current knowledge on exosome biogenesis and release. Cell Mol Life Sci..

[21] Raposo G, Stoorvogel W (2013). Extracellular vesicles: Exosomes, microvesicles, and friends. J Cell Biol..

[22] Colombo M, Raposo G, Théry C (2014). Biogenesis, Secretion, and intercellular interactions of exosomes and other extracellular vesicles. Annu Rev Cell Dev Biol..

[23] Valadi H, Ekstrom K, Bossios A, Sjostrand M, Lee JJ, Lotvall JO (2007). Exosome-mediated transfer of mRNAs and microRNAs is a novel mechanism of genetic exchange between cells. Nat Cell Biol..

[24] Skog J, Würdinger T, van Rijn S, Meijer DH, Gainche L, Curry WT (2008). Glioblastoma microvesicles transport RNA and proteins that promote tumour growth and provide diagnostic biomarkers. Nat Cell Biol..

[25] Al-Nedawi K, Meehan B, Kerbel RS, Allison AC, Rak J (2009). Endothelial expression of autocrine VEGF upon the uptake of tumor-derived microvesicles containing oncogenic EGFR. Proc Natl Acad Sci..

[26] Antonyak MA, Li B, Boroughs LK, Johnson JL, Druso JE, Bryant KL (2011). Cancer cell-derived microvesicles induce transformation by transferring tissue transglutaminase and fibronectin to recipient cells. Proc Natl Acad Sci..

[27] Wang T, Gilkes DM, Takano N, Xiang L, Luo W, Bishop CJ (2014). Hypoxia-inducible factors and RAB22A mediate formation of microvesicles that stimulate breast cancer invasion and metastasis. Proc Natl Acad Sci..

[28] Kanada M, Bachmann MH, Hardy JW, Frimannson DO, Bronsart L, Wang A (2015). Differential fates of biomolecules delivered to target cells via extracellular vesicles. Proc Natl Acad Sci..

[29] Yang C, Guo WB, Zhang WS, Bian J, Yang JK, Zhou QZ (2017). Comprehensive proteomics analysis of exosomes derived from human seminal plasma. Andrology..

[30] Dreux M, Garaigorta U, Boyd B, Décembre E, Chung J, Whitten-Bauer C (2012). Short-range exosomal transfer of viral RNA from infected cells to plasmacytoid dendritic cells triggers innate immunity. Cell Host Microbe..

[31] Simeoli R, Montague K, Jones HR, Castaldi L, Chambers D, Kelleher JH (2017). Exosomal cargo including microRNA regulates sensory neuron to macrophage communication after nerve trauma. Nat Commun..

[32] Jansen F, Yang X, Proebsting S, Hoelscher M, Przybilla D, Baumann K (2014). MicroRNA expression in circulating microvesicles predicts cardiovascular events in patients with coronary artery disease. J Am Heart Assoc..

[33] Rani A, O’Shea A, Ianov L, Cohen RA, Woods AJ, Foster TC (2017). miRNA in circulating microvesicles as biomarkers for age-related cognitive decline. Front Aging Neurosci.

[34] Kobayashi M, Salomon C, Tapia J, Illanes SE, Mitchell MD, Rice GE (2014). Ovarian cancer cell invasiveness is associated with discordant exosomal sequestration of Let-7 miRNA and miR-200. J Transl Med..

[35] Balaj L, Lessard R, Dai L, Cho YJ, Pomeroy SL, Breakefield XO (2011). Tumour microvesicles contain retrotransposon elements and amplified oncogene sequences. Nat Commun..

[36] Thakur BK, Zhang H, Becker A, Matei I, Huang Y, Costa-Silva B (2014). Double-stranded DNA in exosomes: A novel biomarker in cancer detection. Cell Res..

[37] Li Y, Zheng Q, Bao C, Li S, Guo W, Zhao J (2015). Circular RNA is enriched and stable in exosomes: A promising biomarker for cancer diagnosis. Cell Res..

[38] Silva M, Melo SA (2015). Non-coding RNAs in exosomes: new players in cancer biology. Curr Genomics..

[39] Singh Chahar H, Bao X, Casola A (2015). Exosomes and their role in the lfe cycle and pathogenesis of RNA viruses. Viruses..

[40] Meckes DG, Raab-Traub N (2011). Microvesicles and viral infection. J Virol..

[41] Chivero ET, Bhattarai N, Rydze RT, Winters MA, Holodniy M, Stapleton JT (2014). Human pegivirus RNA is found in multiple blood mononuclear cells in vivo and serum-derived viral RNA-containing particles are infectious in vitro. J Gen Virol.

[42] Bukong TN, Momen-Heravi F, Kodys K, Bala S, Szabo G (2014). Exosomes from hepatitis C infected patients transmit HCV infection and contain replication competent viral RNA in complex with Ago2-miR122-HSP90. PLoS Pathog..

[43] Ramakrishnaiah V, Thumann C, Fofana I, Habersetzer F, Pan Q, De Ruiter PE (2013). Exosome-mediated transmission of hepatitis C virus between human hepatoma Huh7.5 cells. Proc Natl Acad Sci.

[44] Longatti A, Boyd B, Chisari FV (2015). Virion-independent transfer of replication-competent hepatitis C virus RNA between permissive cells. J Virol..

[45] Bernard MA, Zhao H, Yue SC, Anandaiah A, Koziel H, Tachado SD (2014). Novel HIV-1 MiRNAs stimulate TNFa release in human macrophages via TLR8 signaling pathway. PLoS One..

[46] Li J, Liu K, Liu Y, Xu Y, Zhang F, Yang H (2013). Exosomes mediate the cell-to-cell transmission of IFN-α-induced antiviral activity. Nat Immunol..

[47] de Martins ST, Kuczera D, Lötvall J, Bordignon J, Alves LR (2018). Characterization of dendritic cell-derived extracellular vesicles during dengue virus infection. Front Microbiol.

[48] Atkin-Smith GK, Paone S, Zanker DJ, Duan M, Phan TK, Chen W (2017). Isolation of cell type-specific apoptotic bodies by fluorescence-activated cell sorting. Sci Rep.

[49] Pariset E, Agache V, Millet A (2017). Extracellular Vesicles: Isolation Methods. Adv Biosyst..

[50] Momen-Heravi F, Balaj L, Alian S, Mantel PY, Halleck AE, Trachtenberg AJ (2013). Current methods for the isolation of extracellular vesicles. Biol Chem..

[51] Nolte-’t Hoen E, Cremer T, Gallo RC, Margolis LB (2016). Extracellular vesicles and viruses: Are they close relatives?. Proc Natl Acad Sci U S A..

[52] Raab-Traub N, Dittmer DP (2017). Viral effects on the content and function of extracellular vesicles. Nat Rev Microbiol..

[53] Telesnitsky A, Wolin SL (2016). The host RNAs in retroviral particles. Viruses..

[54] Grossegesse Marica, Doellinger Joerg, Haldemann Berit, Schaade Lars, Nitsche Andreas (2017). A Next-Generation Sequencing Approach Uncovers Viral Transcripts Incorporated in Poxvirus Virions. Viruses.

[55] Kuhn RJ, Zhang W, Rossmann MG, Pletnev SV, Corver J, Lenches E (2002). Structure of dengue virus: Implications for flavivirus organization, maturation, and fusion. Cell..

[56] Zhang Y, Kaufmann B, Chipman PR, Kuhn RJ, Rossmann MG (2007). Structure of immature West Nile virus. J Virol..

[57] Song H, Li J, Shi S, Yan L, Zhuang H, Li K (2010). Thermal stability and inactivation of hepatitis C virus grown in cell culture. Virol J..

[58] Kostyuchenko VA, Lim EXY, Zhang S, Fibriansah G, Ng TS, Ooi JSG (2016). Structure of the thermally stable Zika virus. Nature..

[59] Malik ZA, Kott KS, Poe AJ, Kuo T, Chen L, Ferrara KW (2013). Cardiac myocyte exosomes: stability, HSP60, and proteomics. AJP Hear Circ Physiol..

[60] Konoshenko MY, Lekchnov EA, Vlassov AV, Laktionov PP (2018). Isolation of extracellular vesicles: general methodologies and latest trends. Biomed Res Int.

[61] Koga Y, Yasunaga M, Moriya Y, Akasu T, Fujita S, Yamamoto S (2011). Exosome can prevent RNase from degrading microRNA in feces. J Gastrointest Oncol..

[62] Kalra H, Adda CG, Liem M, Ang CS, Mechler A, Simpson RJ (2013). Comparative proteomics evaluation of plasma exosome isolation techniques and assessment of the stability of exosomes in normal human blood plasma. Proteomics..

[63] Rider MA, Hurwitz SN, Meckes DG (2016). ExtraPEG: A polyethylene glycol-based method for enrichment of extracellular vesicles. Sci Rep.

[64] Gilfoy FD, Mason PW (2007). West Nile Virus-Induced interferon production is mediated by the double-stranded RNA-dependent protein kinase PKR. J Virol..

[65] Bourne N, Scholle F, Silva MC, Rossi SL, Dewsbury N, Judy B (2007). Early production of type I interferon during West Nile virus infection: role for lymphoid tissues in IRF3-independent interferon production. J Virol..

[66] Kouwaki T, Fukushima Y, Daito T, Sanada T, Yamamoto N, Mifsud EJ (2016). Extracellular vesicles including exosomes regulate innate immune responses to hepatitis B virus infection. Front Immunol..

[67] Liu WJ, Wang XJ, Clark DC, Lobigs M, Hall RA, Khromykh AA (2006). A single amino acid substitution in the West Nile virus nonstructural protein NS2A disables its ability to inhibit alpha/beta interferon induction and attenuates virus virulence in mice. J Virol..

[68] Zhang J, Li S, Li L, Li M, Guo C, Yao J (2015). Exosome and exosomal microRNA: Trafficking, sorting, and function. Genomics, Proteomics Bioinforma..

[69] Kouwaki Takahisa, Okamoto Masaaki, Tsukamoto Hirotake, Fukushima Yoshimi, Oshiumi Hiroyuki (2017). Extracellular Vesicles Deliver Host and Virus RNA and Regulate Innate Immune Response. International Journal of Molecular Sciences.

[70] Schwarz DS, Hutvágner G, Du T, Xu Z, Aronin N, Zamore PD (2003). Asymmetry in the Assembly of the RNAi Enzyme Complex. Cell..

[71] Fukaya T, Iwakawa H, Tomari Y (2014). MicroRNAs block assembly of eIF4F translation initiation complex in *Drosophila*. Mol Cell..

[72] Behm-Ansmant I, Rehwinkel J, Doerks T, Stark A, Bork P, Izaurralde E (2006). mRNA degradation by miRNAs and GW182 requires both CCR4 : NOT deadenylase and DCP1 : DCP2 decapping complexes. Genes Dev..

[73] Shimakami T, Yamane D, Jangra RK, Kempf BJ, Spaniel C, Barton DJ (2012). Stabilization of hepatitis C virus RNA by an Ago2-miR-122 complex. Proc Natl Acad Sci U S A..

[74] Rocha W, Verreault A, Almouzni G, Paik J, Carey M, Workman JL (2007). Switching from repression to activation : microRNAs can up-regulate translation. Science..

[75] Quicke KM, Suthar MS (2013). The innate immune playbook for restricting West Nile virus infection. Viruses..

[76] Hilton L, Moganeradj K, Zhang G, Chen Y-H, Randall RE, McCauley JW (2006). The NPro product of bovine viral diarrhea virus inhibits DNA binding by interferon regulatory factor 3 and targets it for proteasomal degradation. J Virol..

[77] Aqil M, Naqvi AR, Mallik S, Bandyopadhyay S, Maulik U, Jameel S (2014). The HIV Nef protein modulates cellular and exosomal miRNA profiles in human monocytic cells. J Extracell Vesicles..

[78] Arenaccio C, Anticoli S, Manfredi F, Chiozzini C, Olivetta E, Federico M (2015). Latent HIV-1 is activated by exosomes from cells infected with either replication-competent or defective HIV-1. Retrovirology..

[79] Lenassi M, Cagney G, Liao M, Vaupotič T, Bartholomeeusen K, Cheng Y (2010). HIV Nef is secreted in exosomes and triggers apoptosis in bystander CD4+ T cells. Traffic..

[80] Zhou Y, Wang X, Sun L, Zhou L, Ma TC, Song L (2016). Toll-like receptor 3-activated macrophages confer anti-HCV activity to hepatocytes through exosomes. FASEB J..

[81] Roth WW, Huang MB, Konadu KA, Powell MD, Bond VC (2015). Micro RNA in exosomes from HIV-infected macrophages. Int J Environ Res Public Health..

[82] Narayanan A, Iordanskiy S, Das R, Van Duyne R, Santos S, Jaworski E (2013). Exosomes derived from HIV-1-infected cells contain trans-activation response element RNA. J Biol Chem..

[83] Madison MN, Roller RJ, Okeoma CM (2014). Human semen contains exosomes with potent anti-HIV-1 activity. Retrovirology..

[84] Slonchak A, Shannon RP, Pali G, Khromykh AA (2015). Human microRNA miR-532-5p exhibits antiviral activity against West Nile virus via suppression of host genes SESTD1 and TAB3 required for virus replication. J Virol..

[85] Slonchak A, Hussain M, Torres S, Asgari S, Khromykh AA (2014). Expression of mosquito microRNA Aae-miR-2940-5p is downregulated in response to West Nile virus infection to restrict viral replication. J Virol..

[86] Smith JL, Grey FE, Uhrlaub JL, Nikolich-Zugich J, Hirsch AJ (2012). Induction of the cellular microRNA, Hs_154, by West Nile virus contributes to virus-mediated apoptosis through repression of antiapoptotic factors. J Virol..

[87] Chugh PE, Damania BA, Dittmer DP (2014). Toll-like receptor-3 is dispensable for the innate microRNA response to West Nile virus (WNV). PLoS One..

[88] Smith JL, Jeng S, McWeeney SK, Hirsch AJ (2017). A MicroRNA Screen identifies the Wnt signaling pathway as a regulator of the interferon response during flavivirus infection. J Virol..

[89] Schurch NJ, Schofield P, Gierliński M, Cole C, Sherstnev A, Singh V (2016). How many biological replicates are needed in an RNA-seq experiment and which differential expression tool should you use?. Rna..

[90] Thounaojam MC, Kaushik DK, Kundu K, Basu A (2014). MicroRNA-29b modulates Japanese encephalitis virus-induced microglia activation by targeting tumor necrosis factor alpha-induced protein 3. J Neurochem..

[91] Choi JE, Hur W, Kim JH, Li TZ, Lee EB, Lee SW (2014). MicroRNA-27a modulates HCV infection in differentiated hepatocyte-like cells from adipose tissue-derived mesenchymal stem cells. PLoS One..

[92] Zhang L, Chen X, Shi Y, Zhou B, Du C, Liu Y (2014). miR-27a suppresses EV71 replication by directly targeting EGFR. Virus Genes..

[93] Machitani M, Sakurai F, Wakabayashi K, Nakatani K, Tachibana M, Mizuguchi H (2017). MicroRNA miR-27 inhibits adenovirus infection by supressing the expression of SNAP2 and TXN2. J. Virol..

[94] Zheng Q, Hou J, Zhou Y, Yang Y, Cao X (2016). Type I IFN–inducible downregulation of microRNA-27a feedback inhibits antiviral innate response by upregulating Siglec1/TRIM27. J Immunol..

[95] Wang Y, Wang C-M, Jiang Z-Z, Yu X-J, Fan C-G, Xu F-F (2015). MicroRNA-34c targets TGFB-induced factor homeobox 2, represses cell proliferation and induces apoptosis in hepatitis B virus-related hepatocellular carcinoma. Oncol Lett..

[96] Jia X, Bi Y, Li J, Xie Q, Yang H, Liu W (2015). Cellular microRNA miR-26a suppresses replication of porcine reproductive and respiratory syndrome virus by activating innate antiviral immunity. Sci Rep.

[97] Gao S, Li J, Song L, Wu J, Huang W (2017). Influenza A virus-induced downregulation of miR-26a contributes to reduced IFNα/β production. Virol Sin..

[98] Youssef OA, Safran SA, Nakamura T, Nix DA, Hotamisligil GS, Bass BL (2015). Potential role for snoRNAs in PKR activation during metabolic stress. Proc Natl Acad Sci..

[99] Scott MS, Ono M (2011). From snoRNA to miRNA: Dual function regulatory non-coding RNAs. Biochimie..

[100] Zhong F, Zhou N, Wu K, Guo Y, Tan W, Zhang H (2015). A SnoRNA-derived piRNA interacts with human interleukin-4 pre-mRNA and induces its decay in nuclear exosomes. Nucleic Acids Res..

[101] Manokaran G, Finol E, Wang C, Gunaratne J, Bahl J, Ong EZ (2015). Dengue subgenomic RNA binds TRIM25 to inhibit interferon expression for epidemiological fitness. Science..

[102] Fredericksen BL, Keller BC, Fornek J, Katze MG, Gale M (2008). Establishment and maintenance of the innate antiviral response to West Nile virus involves both RIG-I and MDA5 signaling through IPS-1. J Virol..

[103] Morales DJ, Lenschov DJ (2013). The antiviral activities of ISG15. J Mol Biol..

[104] Liu WJ, Wang XJ, Mokhonov VV, Shi P-Y, Randall R, Khromykh AA (2005). Inhibition of interferon signaling by the New York 99 strain and Kunjin subtype of West Nile virus involves blockage of STAT1 and STAT2 activation by nonstructural proteins. J Virol..

[105] Setoh Y, Periasamy P, Peng N, Amarilla A, Slonchak A, Khromykh A (2017). Helicase domain of West Nile virus NS3 protein plays a role in inhibition of type I interferon signalling. Viruses..

[106] Khromykh AA, Kenney MT, Edwin G, Westaway EG (1998). trans-Complementation of Flavivirus RNA Polymerase Gene NS5 by Using Kunjin Virus Replicon-Expressing BHK Cells. J Virol..

[107] Chen C, Ridzon DA, Broomer AJ, Zhou Z, Lee DH, Nguyen JT (2005). Real-time quantification of microRNAs by stem-loop RT-PCR. Nucleic Acids Res..

[108] Le Carré J, Lamon S, Léger B (2014). Validation of a multiplex reverse transcription and pre-amplification method using TaqMan® MicroRNA assays. Front Genet.

[109] Dobin A, Davis CA, Schlesinger F, Drenkow J, Zaleski C, Jha S (2013). STAR: Ultrafast universal RNA-seq aligner. Bioinformatics..

[110] Anders S, Pyl PT, Huber W (2015). HTSeq-A Python framework to work with high-throughput sequencing data. Bioinformatics..

[111] Robinson MD, McCarthy DJ, Smyth GK (2010). edgeR: a Bioconductor package for differential expression analysis of digital gene expression data. Bioinformatics..

[112] Fan Y, Siklenka K, Arora SK, Ribeiro P, Kimmins S, Xia J (2016). miRNet - dissecting miRNA-target interactions and functional associations through network-based visual analysis. Nucleic Acids Res.

[113] Huang DW, Sherman BT, Lempicki RA (2009). Systematic and integrative analysis of large gene lists using DAVID bioinformatics resources. Nat Protoc..

[114] Walter W, Sánchez-Cabo F, Ricote M (2015). GOplot: An R package for visually combining expression data with functional analysis. Bioinformatics..

