# Supplementary figures and images for: West Nile virus infection and interferon alpha treatment alter the spectrum and the levels of coding and noncoding host RNAs secreted in extracellular vesicles
Source: BMC Genomics. 2019 Jun 10;20:474. doi: 10.1186/s12864-019-5835-6 (PMC6558756; doi:10.1186/s12864-019-5835-6)

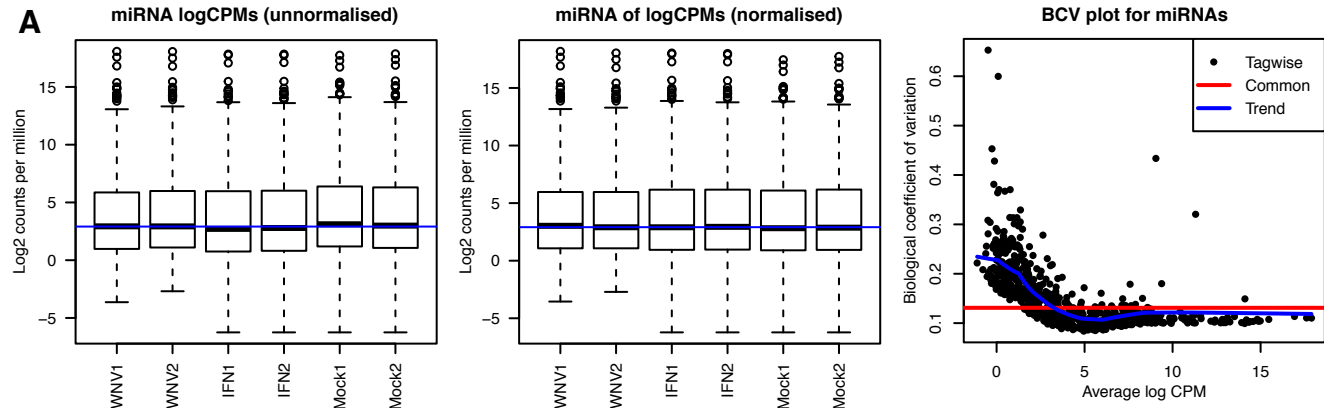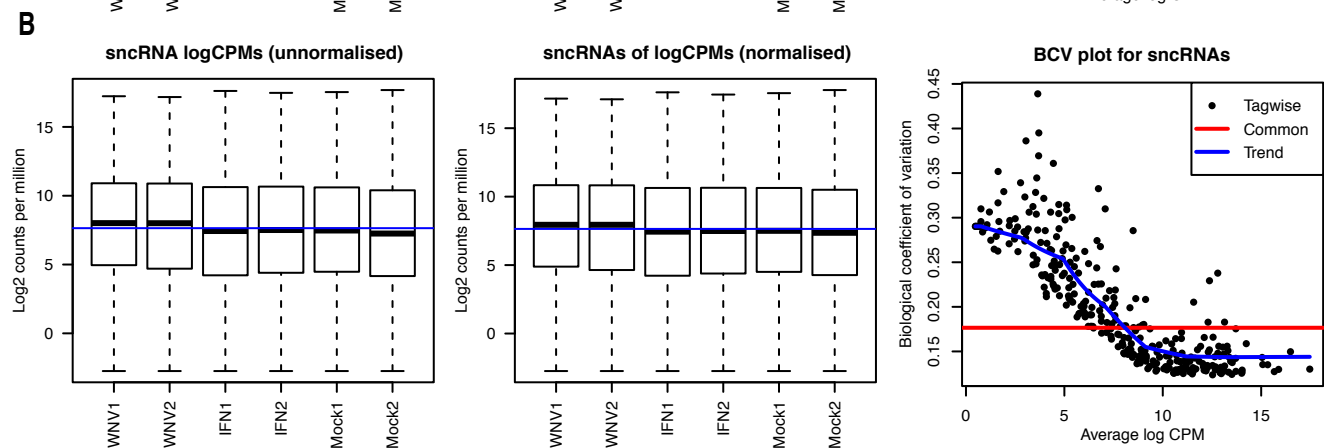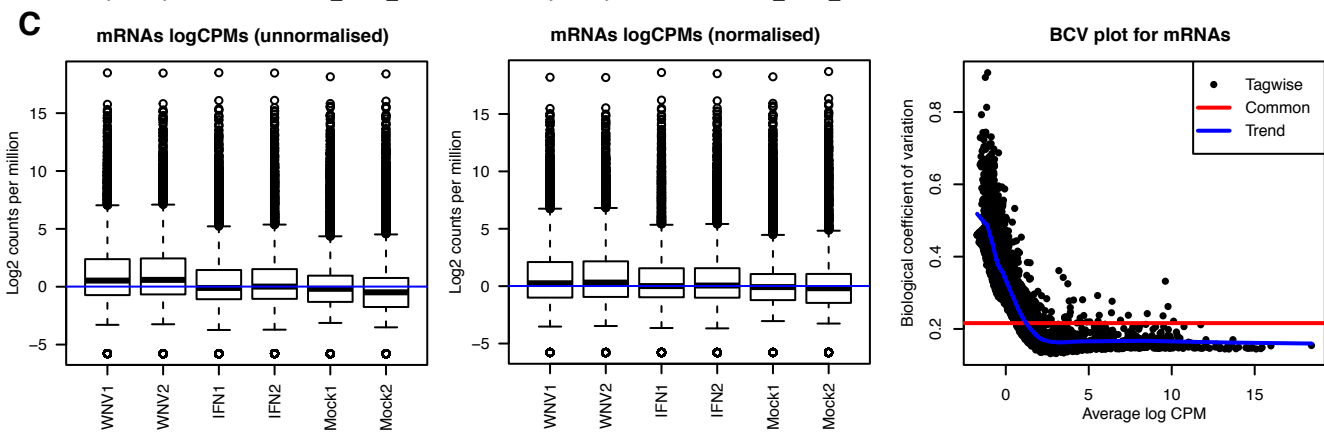

Supplement: Supplementary file 1 — Figure S1. Characterization of RNASeq datasets. Box plots represent abundance distribution for all RNAs in datasets before and after normalisation and BCV plots show variations between the biological replicates after normalisation for EV miRNAs (A), sncRNAs (B) and mRNAs (C). (PDF 296 kb) [file 12864_2019_5835_MOESM1_ESM.pdf]

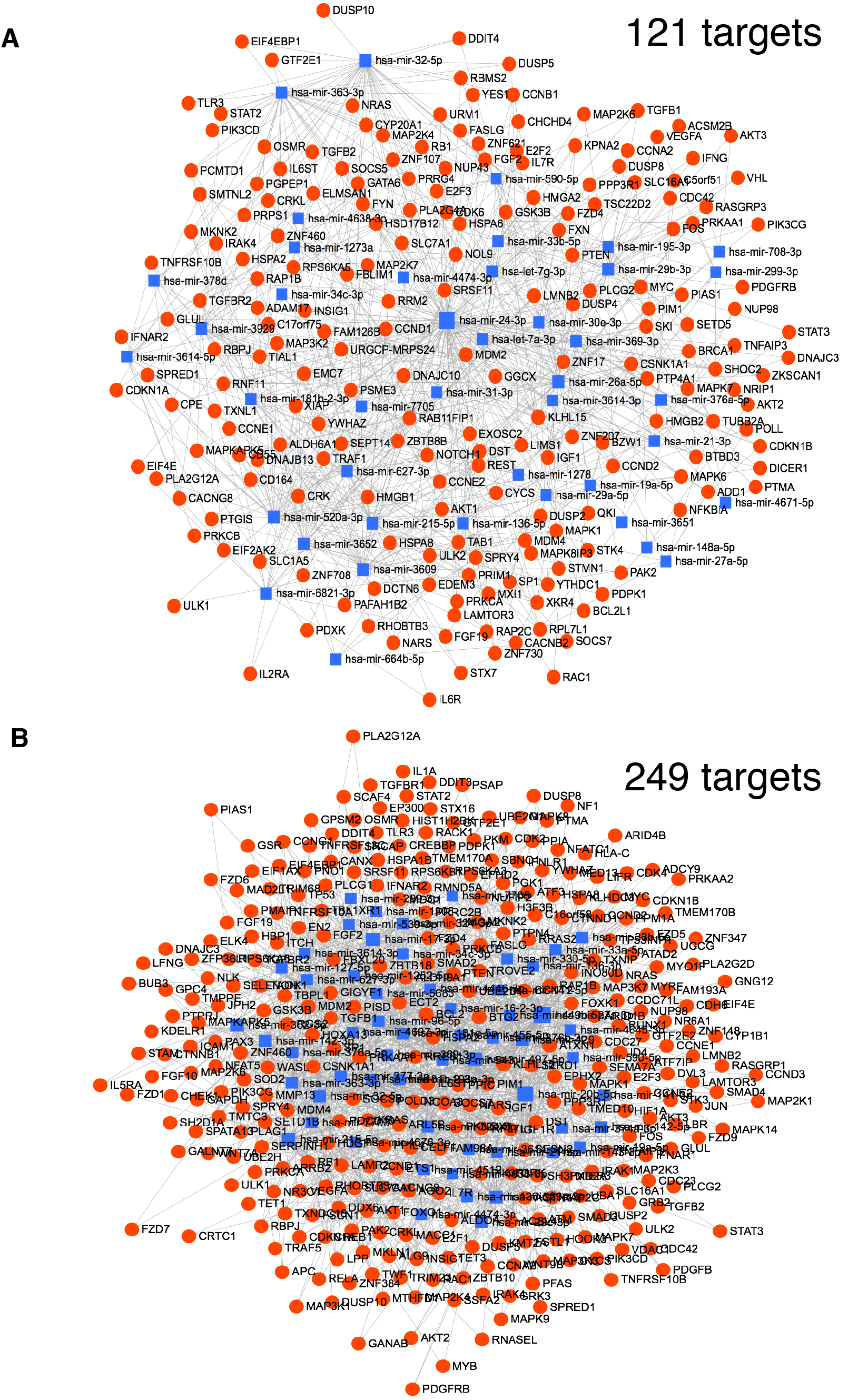

Supplement: Supplementary file 2 — Figure S2. Targets of miRNAs differentially incorporated into EVs from WNV-infected (A) and IFN-treated (B) A549 cells associated with virus-related pathways. To obtain the networks lists of experimentally validated mRNA-miRNA interactions for each miRNA dataset were extracted from miRNet database, then mRNAs targeted by<2 miRNAs and miRNAs targeting <2 mRNAs were removed from the list by applying the filter with “degree cut-off” =1 in miRNet software. The resulted interactions were used to reconstruct the networks, which then were subjected to functional exploration in which enriched KEGG pathways associated with the network components were identified and subnetworks associated with KEGG pathways related to virus-host interactions were extracted. (TIF 6758 kb) [file 12864_2019_5835_MOESM2_ESM.tif]

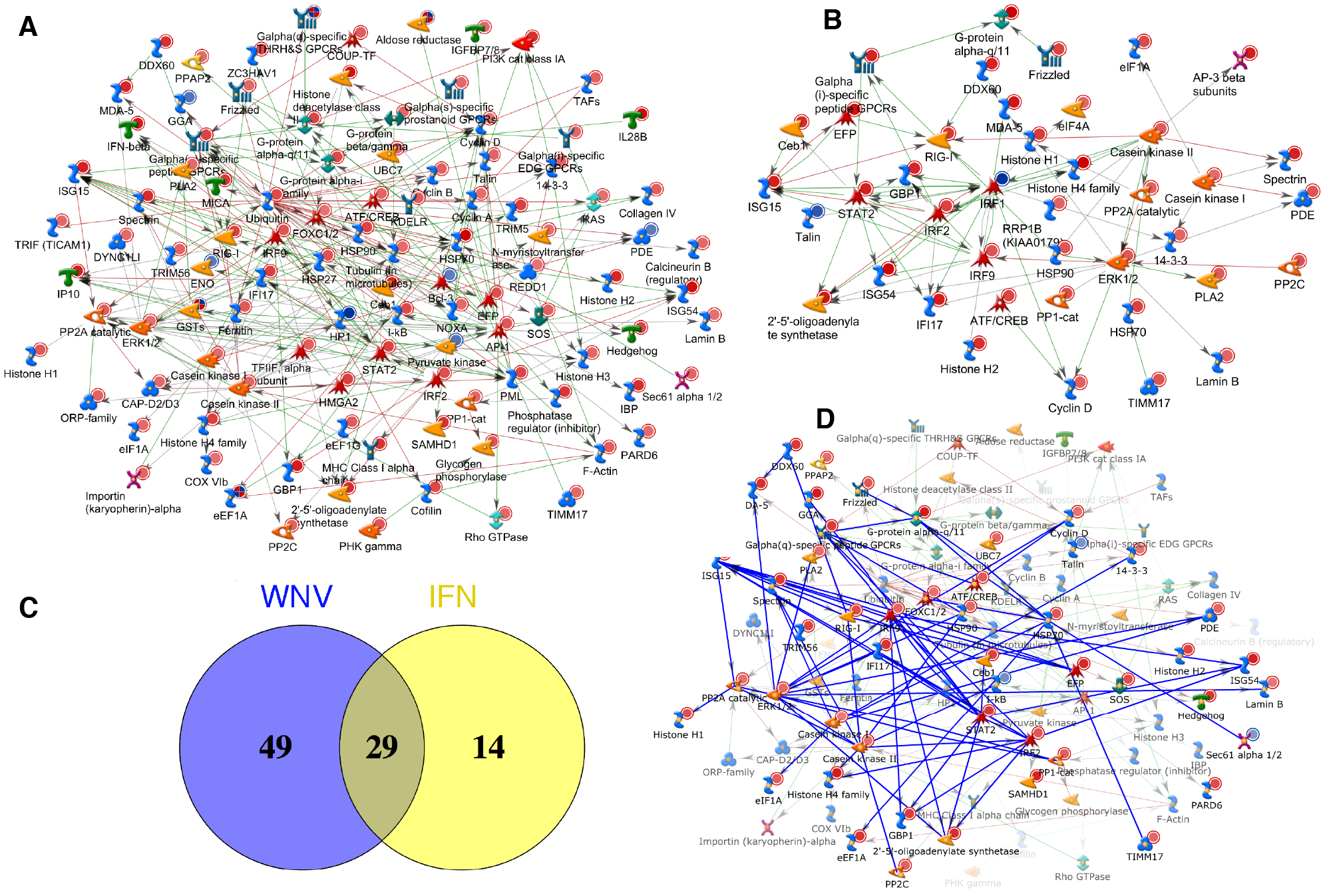

Supplement: Supplementary file 3 — Figure S3. Comparison of the antiviral pathway-associated networks reconstructed from mRNAs with increased abundance in EVs from WNV-infected or IFN-alpha-treated cells. (A, B) Simulated networks of the components of antiviral pathways encoded by mRNAs increased in EVs secreted from WNV-infected (A) or IFN-alpha-treated (B) cells. (C) Numbers of unique and common network nods associated with antiviral response that are enriched in EVs secreted from WNV-infected and IFN-alpha-treated cells. (D) Results of superimposing (B) to (A) indicating which of WNV-induced EV mRNAs and antiviral pathways overlap with those induced by IFN-alpha treatment. Red (induction) and blue (repression) circles near the nods indicate the changes in mRNA level in WNV EVs (A) or IFN-alpha EVs (B, D) with the intensity of the colouring indicating the level of change. Red edges indicate inhibitory and green edges indicate stimulatory interactions between network components, grey edges indicate that the effect of interaction is not known; symbols of the nods indicate functional category of the network component (https://portal.genego.com/legends/MetaCoreQuickReferenceGuide.pdf). To reconstruct the networks, list of the differentially incorporated mRNAs with log2-fold change values and P-values were submitted to MetaCore software and network reconstruction was performed using “direct interactions” algorithm, which assigns network edges to the experimentally validated interactions between the genes present in manually curated MetaCore database. Then MetaCore filters were applied to the resulted large networks, which allows selection of GeneGo terms from the lists of terms significantly associated with the genes present in the network. Terms related to antiviral response and sensing of viral RNA were selected from the list and genes that are not assigned to these terms were hidden from the network. The resulted simplified networks, which only contains components directly associated with antiviral a [file 12864_2019_5835_MOESM3_ESM.tif]
